# Genomic insights and the conservation potential of captive breeding: The case of Chinese alligator

**DOI:** 10.1126/sciadv.adm7980

**Published:** 2025-04-02

**Authors:** Tao Pan, Jiashun Miao, Ke Sun, Haitao Nie, Nicholas M. Luscombe, Wengang Li, Song Zhang, Liuyang Yang, Huan Wang, Yongkang Zhou, Genjun Tu, Yilin Shu, Baowei Zhang, Xiaobing Wu

**Affiliations:** ^1^ College of Life Sciences, Anhui Normal University, Wuhu, Anhui 241000, China.; ^2^ Anhui Provincial Key Laboratory of Biodiversity Conservation and Ecological Security in the Yangtze River Basin, Wuhu, Anhui 241000, China.; ^3^ Genomics and Regulatory Systems Unit, Okinawa Institute of Science and Technology Graduate University, Onna-son, Okinawa 904-0495, Japan.; ^4^ Xianghu Laboratory, Hangzhou, Zhejiang 311231, China.; ^5^ Anhui Research Center of Chinese Alligator Reproduction, Xuancheng, Anhui 242000, China.; ^6^ School of Life Sciences, Anhui University, Hefei, Anhui 230601, China.

## Abstract

Despite 40 years of conservation of the critically endangered Chinese alligator (*Alligator sinensis*), the genomic underpinnings of its status remained uncharted. Genome sequencing data of 244 individuals uncovered relatively low overall genomic diversity/heterozygosity and long runs of homozygosity, with captive populations exhibiting higher heterozygosity and smaller inbreeding coefficients compared to wild individuals. The decreased level of inbreeding in the captive population demonstrates the contribution of the large captive breeding population. The estimated recent effective population size was around a few dozen. To combat challenges of inbreeding depression and reduced adaptability, we used genome-wide SNP-based kinship analysis on captive populations to enable a genome-informed breeding program that minimizes inbreeding. Long-term field monitoring revealed that the Chinese government greatly advanced the conservation of *A. sinensis* through conservation measures and reintroduction programs. Our research enriches the understanding of the Chinese alligator’s genetic landscape, offering invaluable genomic resources for breeding and conservation strategies.

## Teaser

Captive breeding enhances the conservation of the Chinese alligator by promoting population recovery and genetic diversity.

## INTRODUCTION

Threatened and endemic species, particularly those critically endangered, face challenges due to small, fragmented habitats and environmental changes such as habitat destruction, climate change, and disease (*1*). These challenges are exacerbated by genetic factors such as loss of diversity, increased inbreeding, and genetic drift, which diminish species fitness and viability (*2*–*5*). The degree of these genetic effects varies across populations and species due to different demographic histories, life-history traits, and environmental sensitivities (*6*). Assessing the degree of genomic erosion, inbreeding, and genetic load is a crucial, effective conservation strategy.

The conservation of endangered germplasm focuses on reducing inbreeding and homozygosity to enhance fitness (*7*). Inbreeding can cause the manifestation of (partially) recessive deleterious alleles or, in rare cases, reduce fitness due to the loss of heterozygotic advantage, which causes the detrimental effects on a broad range of traits, individual fitness, and population viability (*8*). Continued decline in animal population size (*9*) and global habitat fragmentation (*10*) are likely to accelerate inbreeding. Thus, it is important to develop a detailed understanding of genetic causes of population size decline and fitness consequences to develop conservation strategies. However, the basic features of inbreeding depression in wild or domestic populations still need to be further studied, such as the depth of inbreeding depression, action process in different life-history stages, and deleterious effects of gene-driven fitness reduction (*4*).

With its critically endangered status and charismatic nature, the Chinese alligator (*Alligator sinensis*) has received considerable conservation attention. In the late 1970s and early 1980s, B. Chen from Anhui Normal University, in collaboration with American scholar M. E. Watanabe and Z. Huang from Chinese Academy of Sciences, initiated a survey of the wild population of Chinese alligators in the Yangtze River region (*11*). This collaboration marked the beginning of ecological research on the Chinese alligator population in China. Building upon joint Sino-American surveys, the Yangtze Alligator Breeding Research Center and the Anhui Province Yangtze Alligator Nature Reserve (provincial level) were established successively in Xuancheng, Anhui Province, during 1982–1983. The nature reserve is situated at the junction of the southern Anhui mountainous region and the lower Yangtze River plain, encompassing Xuancheng, Langxi, Guangde, Jingxian, and Nanling. The successful large-scale artificial breeding of Chinese alligators was achieved in 1984. In 1986, the nature reserve was officially upgraded to a national-level reserve, with a total area of 43,300 ha at that time. In response to the distribution of wild Chinese alligator populations, 5 protection stations and 13 protection points were established. The nature reserve meticulously adheres to the Wildlife Protection Law, Regulations on Nature Reserves, and Management Measures for Forest and Wildlife Type Nature Reserves. Emphasizing protection as its foundation, the reserve has made notable and effective efforts in infrastructure development, personnel training, monitoring and research of Chinese alligators, community education, and international exchanges and cooperation. These efforts have contributed to the protection of Chinese alligators and their habitats (*11*).

This study highlights over four decades of conservation efforts in the Anhui Chinese Alligator National Nature Reserve (ACANNR), emphasizing great progress in breeding and habitat restoration. We present a comprehensive genetic assessment of the Chinese alligator, including a chromosome-level reference genome and whole-genome resequencing of different populations. Our findings shed light on genetic diversity, inbreeding levels, population relationships, and demographic history, contributing to the conservation and breeding planning of this critically endangered species.

## RESULTS

### A chromosome-scale genome assembly of Chinese alligator

**Fig. 1.**
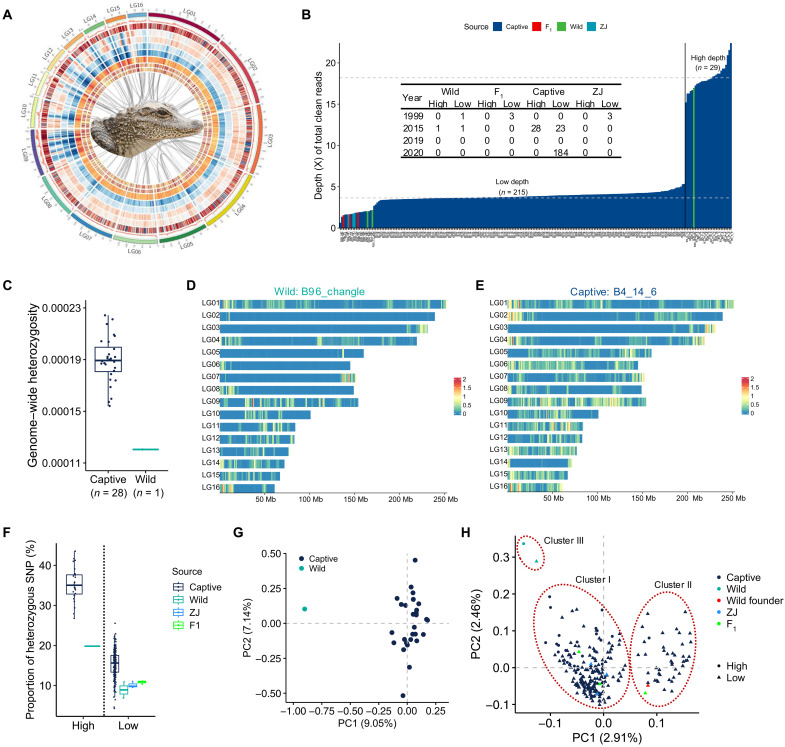
Genome assembly and population genomic study of Chinese alligator revealing genetic diversity/heterozygosity and population structure. (**A**) Chromosome-scale genome assembly of Chinese alligator. Circos graph shows characteristics of the assembly. From outer circle to inner ring: guanine-cytosine (GC) content (100-kb window), gene density (1-Mb window), long interspersed nuclear element density (1-Mb window), Terminal Inverted Repeats (TIR) density (1-Mb window), *Penelope*-like elements density (1-Mb window), long-tandem repeats (1-Mb window), large retrotransposon derivative density (1-Mb window), gene expression profile in a male (ID: MD31) and a female sample (ID: FD41). (**B**) Sequencing depth distribution of 215 low-depth (~3.65×) and 29 normal-depth (~18.19×) sequencing individuals. Inserted table summaries sample numbers for each group. (**C**) Genome-wide heterozygosity comparison between the normal-depth sequenced wild individual (sample ID: B96_changle) and the captive population (*n* = 28). (**D**) Genomic distribution of heterozygous SNPs of the wild individual, B96_change. The heterozygous SNPs per kilobyte was calculated using nonoverlap 1-Mb window. (**E**) Genomic distribution the captive individual, B4_24_6. (**F**) Percentage of heterozygous SNPs for 215 low-depth and 29 normal-depth sequenced individuals, color-coded by the source. (**G**) Principal components (PC) analysis (PCA) for 29 normal-depth sequenced individuals by using 1,001,965 genome-wide SNPs. (**H**) PCA for 215 low-depth and 29 normal-depth sequenced individuals based on 402,440 SNPs. Triangles and points were used to represent individuals sequenced at low depth and normal depth, respectively.

The genome size of our sequencing Chinese alligator (*A. sinensis*) estimated by *k*-mer statistic was about 2.40 Gb, with a heterozygosity of about 0.05% (fig. S1). About 230.95-Gb corrected Nanopore reads, with read N50 of 25.92 kb, was used for de novo assembly (tables S1 and S2). A 2.26-Gb preliminary contig assembly, with contig N50 length of 35.2 Mb, was obtained, accounting for 94.17% of the *k*-mer–estimated genome size. Through Hi-C scaffolding, a total of 2258.54 (99.78%)–Mb contigs were clustered into 16 linkage groups, which happens to correspond with 16 pseudochromosomes (2*n* = 32) (Fig. 1A and tables S3 and S4) (*12*). Last, we obtained a chromosome-level, high-quality Chinese alligator assembly (2.26 Gb) with a scaffold N50 length of 154.26 Mb, providing a genomic resource to assist further study of the Chinese alligator (Fig. 1A). We recalled 94.3% (3724) Benchmarking Universal Single-Copy Orthologue (BUSCO) conserved core genes in tetrapoda lineage dataset from the current genome assembly, which was a little higher compared with the previously released scaffold-level assembly (92.8%) (table S5) (*13*). In addition, 442 of 458 (96.51%) conserved core genes in eukaryotes from the Core Eukaryotic Genes Mapping Approach (CEGMA) database were recalled from our genome assembly (*14*). We mapped 44 DNA sequencing (DNA-seq) and 32 RNA sequencing (RNA-seq) datasets against the current and previously released assemblies (*13*), respectively. All datasets showed exceptionally high alignment rates for both assemblies and with the current assembly showing a slightly higher overall alignment rate (98.41%) for the DNA-seq datasets (tables S6 and S7). Pairwise genome comparison between the current and previous assemblies revealed about 1.91 million single-nucleotide polymorphisms (SNPs) and 0.57 million small insertions and deletions (InDels), indicating low genetic differentiation between the two sequenced individuals (table S8).

We identified 950.25-Mb (41.98%) repeat sequences in our genome assembly. A total of 21,237 protein-coding, 226 microRNA, 176 ribosomal RNA (rRNA), 879 transfer RNA (tRNA), 242 small nuclear RNA, and 226 small nucleolar RNA genes were predicted (tables S9 and S10). The majority of the predicted protein-coding genes, specifically 20,614 (97.07%), were successfully annotated by functional databases (table S11).

### Genetic diversity (heterozygosity) difference between wild and captive population

To investigate the genomic heterozygosity of wild and captive populations, we sequenced the genome of 244 individuals. Of these, 29 individuals were sequenced at an average depth of about 18.19×, while the remaining 215 individuals were sequenced at a lower depth of about 3.65× (Fig. 1B and table S12). Among the group of 29, one individual (sample ID: B96_changle) resided in the wild, and the other 28 were captive-bred, primarily third-generation offspring of wild-born individuals. Across these 29 individuals, we identified 1,344,405 SNPs and 641,922 InDels, with an overall genotyping rate of 97.62%. Notably, 57,249 SNPs differed between the wild individual and the captive population, highlighting their potential utility in identifying wild Chinese alligators (data S1). We conducted sorting intolerant from tolerant (SIFT) score predictions for these 29 individuals. Among the 25,829 SNPs with SIFT predictions, 4013 (15.54%) SNPs were predicted to be deleterious (SIFT score < 0.05), with most being nonsynonymous. The average number of deleterious mutations in the captive population (*n* = 28) did not differ from that in the wild individual (B96_changle).

The average genome-wide heterozygosity (H) across all 29 individuals was about 1.87 × 10^–4^ ± 2.17 × 10^−5^, with captive population (*n* = 28) displaying an average H of 1.90 × 10^−4^ ± 1.78 × 10^−5^ and the wild individual (sample ID: B96_changle) showing an H of around 1.20 × 10^−4^ (table S13). The genome-wide heterozygosity of captive population was significantly higher than that of wild individual (*t* test, *P* value < 5.51 × 10^−16^) (Fig. 1, C to E). Our findings demonstrated that the average H of 1.20 × 10^−4^ observed in the wild individual is consistent with a recent study that analyzed 23 Chinese alligators (*15*). However, the captive population in our study, consisting of 28 individuals, displayed a significantly higher average H value than the previous study.

By integrating the data from 215 low-depth sequenced individuals with the 29 individuals, we got 1,650,931 SNPs after filtering. The missing genotyping rates range from 0.48 to 80.55% for each individual, with an average missing rate of 0.61% for the 29 individuals and 20.73% for the 215 low-depth sequenced individuals. We filtered these SNPs located on 16 pseudochromosomes with a missing rate < 20% and a minor allele frequency (MAF) > 0.05, resulting in 402,440 retained SNPs. The average missing rate for the 215 low-depth individuals then decreased to ~15% (fig. S2). Subsequently, we performed genotype imputation for this variant dataset. The percentage of heterozygous SNPs for the 215 low-depth individuals (15.18 ± 3.47) was lower than that of the 29 normal-depth individuals (34.70 ± 4.90), indicating that low-depth sequencing could lead to underestimating of genomic heterozygosity (Fig. 1F). However, we believe that when the sequencing depth of different individuals is similar, but the estimated heterozygosity difference is very large, this difference should be considered true.

Within these 215 low-depth sequenced individuals, the average proportion of heterozygous SNPs of the captive population was about 15.38 ± 3.37% (Fig. 1F). The average proportion of three captive individuals from Zhejiang Province (ZJ) and three F_1_ individuals was about 10.11 ± 0.78 and 10.80 ± 0.40%, respectively (Fig. 1F). Notably, two wild individuals (sample IDs: R58_CL_wild and R99_wild) had proportions of about 4.74 and 6.71%, which were lower than the average value of the captive population (Fig. 1F).

Together, the genome-wide heterozygosity of the three wild individuals collected at different periods (1999 and 2015) was lower than that of most investigated captive individuals (*n* = 241) in the current conservation area in China. This indicates that artificial breeding increased the heterozygosity, which may be conducive to the adaptive survival of individuals for the Chinese alligator and reduced the probability of recessive homozygous disease-causing genes.

### Population structure

Principal components analysis (PCA) based on the 1,001,965 genome-wide SNPs of 29 normal-depth sequenced individuals distinguished the wild individual (sample ID: B96_changle) from the captive population (*n* = 28) in the first dimension (Fig. 1G). In addition, Identity-By-Decent (IBD) analysis revealed no relatedness between the wild individual and the 28 captive individuals. Phylogenetic analysis based on the SNPs confirmed this result, as individuals from the same clutch were clustered together (fig. S3).

To assess genetic differentiation among populations, we combined the data from 215 low-depth sequenced individuals with data from the 29 normal-depth sequenced individuals. This integration resulted in 53,972 SNPs after linkage disequilibrium (LD) pruning, which were used for PCA. The PCA results revealed distinct genetic clustering patterns. Notably, two wild individuals (sample IDs: R58_CL_wild and B96_changle), both captured in Changle in 2015, formed a separate genetic cluster, indicating notable genetic differentiation from other populations and high genetic resource value (Fig. 1H and fig S4). The remaining 242 individuals were grouped into two distinct clusters (clusters I and II) by PC1, which accounted for 2.91% of the variance (Fig. 1H). All three individuals from ZJ were assigned to cluster I, while R99_wild, a wild founder individual captured in 1999 for the original population construction in ACANNR, fell into cluster II. The difference between clusters I and II likely reflect the different genetic backgrounds of the wild founder individuals in the establishment group.

The two wild individuals (sample IDs: B96_changle and R58_CL_wild) captured in 2015 along with the individual captured in 1999 (sample ID: R99_wild) exhibited notable differences in their genetic backgrounds (Fig. 1H). Furthermore, three F_1_ generation individuals from the captured wild population were also assigned to two genetic clusters (Fig. 1H). These findings directly indicate that the genetic differentiation within wild population should be higher than that of the current captive populations.

**Fig. 2.**
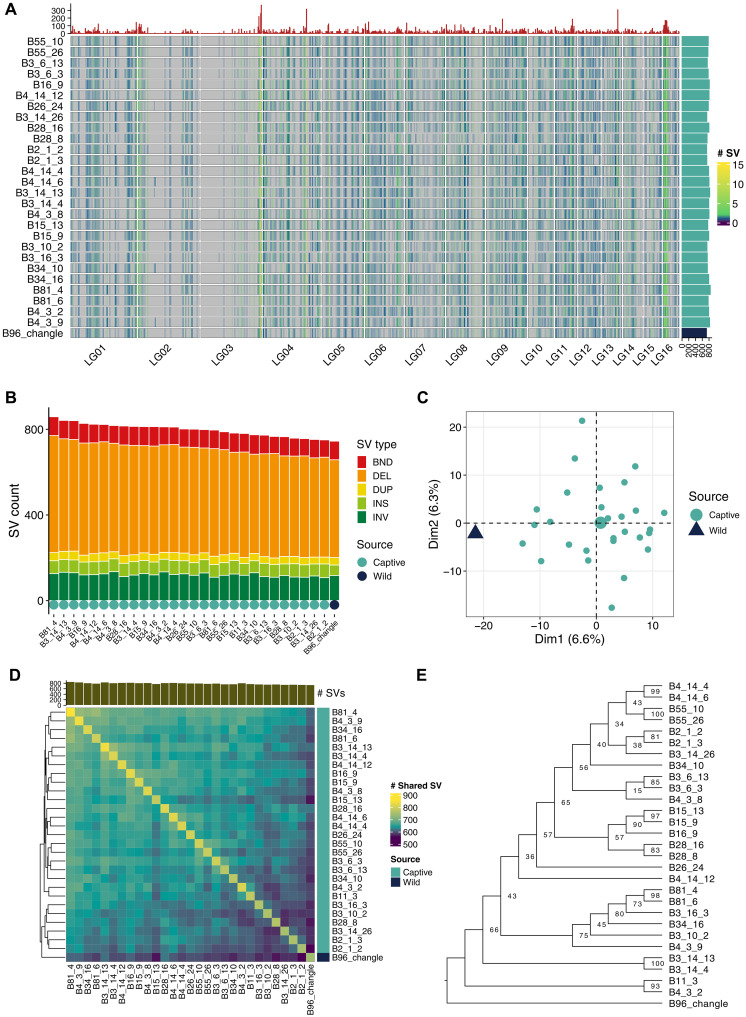
SVs of Chinese alligator. (**A**) Genomic distributions of SVs across 29 normal-depth sequenced individuals. The heatmap shows the number of SV within each nonoverlapped 1-Mb sliding window. The top barplot shows the cumulative SV number across 29 normal-depth sequenced individuals. The barplot on the right side shows the total number of SV across 19 chromosomes of each individual. (**B**) Barplot shows the total number of SVs called in each sample. Different colors were used to indicate different SV types. (**C**) PCA based on the SVs of 29 normal-depth sequenced individuals. (**D**) Heatmap shows the number of SVs shared between each two samples. (**E**) Maximum likelihood phylogeny tree was reconstructed using the SVs. The bootstrap values are indicated on the node.

Structural variations (SVs) were also analyzed to explore the population structure (Fig. 2). A total of 1258 SVs were identified across the 29 normal-depth sequenced individuals, with deletions being the most abundant type (Fig. 2, A and B). PCA based on the SVs revealed notable differences between the wild individual and captive population (Fig. 2C). Individuals from the same clutch tended to share more common SVs, consistent with the results of maximum likelihood phylogeny based on SVs (Fig. 2, D and E) and the SNP-based analysis.

### Kinship analyses of individuals within populations

**Fig. 3.**
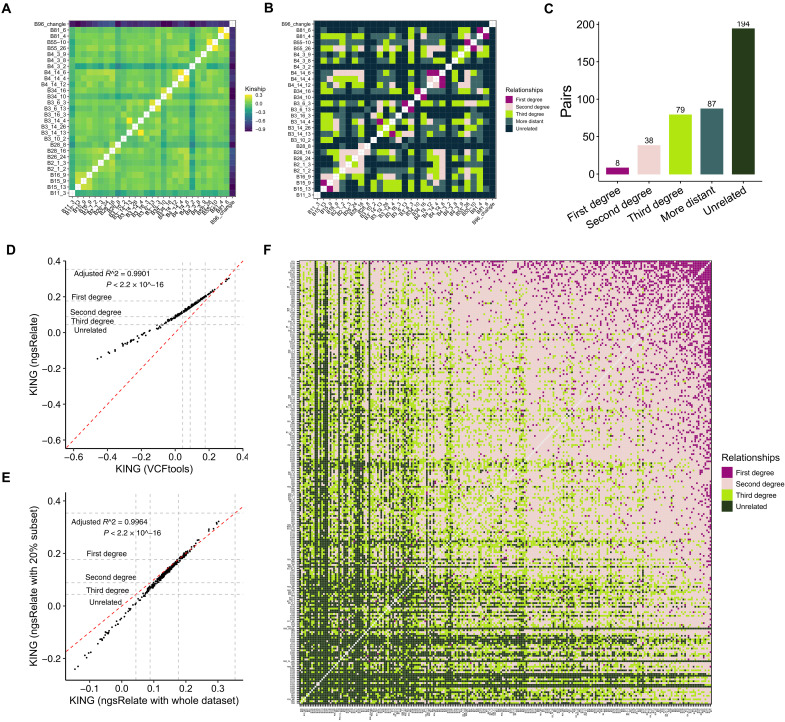
Kinship analyses of individuals within populations. (**A**) Kinship coefficient estimated by KING software for 29 normal-depth sequenced individuals. The color gradient has been scaled according to the kinship coefficient. A negative kinship coefficient estimation indicates an unrelated relationship. (**B**) Degrees of family relationships estimated by KING software for 29 normal-depth sequenced individuals. (**C**) Number of genetically determined relationships by degree of relatedness for 29 normal-depth sequenced individuals. (**D**) Kinship coefficient comparison between two software (NgsRelate and VCFtools) for 29 normal-depth sequenced individuals. The *x* axis shows the kinship coefficient calculated by NgsRelate. The *y* axis shows the kinship coefficient calculated by VCFtools. The dash lines indicate the range of each degree. (**E**) Kinship coefficient comparison between whole dataset (~20×) and 20% down sampling (~4×) for the 29 normal-depth sequenced individuals estimated by NgsRelate. The *x* axis shows the kinship coefficient estimates using whole dataset. The *y* axis shows the estimates using 20% downsampled dataset. (**F**) Relatedness inference for 29 normal-depth and 215 low-depth sequenced individuals using NgsRelate, a kinship inference tool for low-coverage NGS data.

Identical-By-Descent analysis of 29 normal-depth sequenced individuals disclosed eight pairs with a first-degree relationship, which was basically consistent with previously known information about whether these pairs were born in the same clutch (Fig. 3, A to C), whereas 194 relationship pairs were unrelated (Fig. 3C). To assess the genetic relationships within captive populations, we performed relatedness inference for both 215 low-depth and 29 normal-depth sequenced individuals using NgsRelate (*16*), a tool that infers relatedness using genotype likelihoods (GLs) instead of genotypes from low-depth sequencing data. Previous studies have shown that the kinship coefficient estimation depends on both the software and sequencing depth, making accurate relatedness inference challenging with low-depth sequencing (*17*, *18*). We conducted a comparison between NgsRelate and VCFtools using the 29 individuals sequenced with normal depth and observed that NgsRelate tended to overestimated relatedness compared with VCFtools (Fig. 3D). Furthermore, we found that low-depth sequencing could lead to an underestimation of unrelated and distant relatedness when using NgsRelate, as evidenced by comparing the kinship coefficients between downsampling to 20% of the total sequences (~4×) and the entire dataset of the 29 normal-depth sequenced individuals (Fig. 3E). Overall, the relatedness inference of 244 individuals by NgsRelate revealed a substantial proportion of second-degree relationship pairs, which may include half-siblings, double cousins, uncles, aunts, nephews and nieces, indicating the existence of a higher proportion of close relationships within captive populations (Fig. 3F and fig. S5). In addition, two wild individuals captured from Changle, Anhui Province in 2015 were classified as first-degree relationship. Approximately 22% pairs were identified as unrelated, suggesting their great potential use for breeding purposes (Fig. 3F).

### Inbreeding history of wild and captive populations

**Fig. 4.**
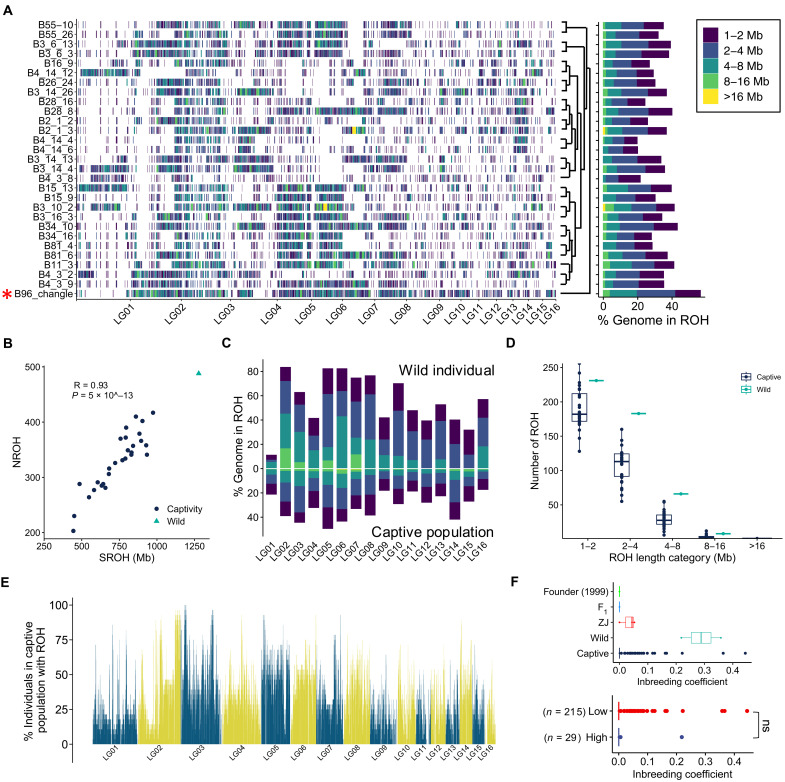
ROH across 29 individuals and inbreeding coefficient of 244 individuals. (**A**) Genomic distribution of ROH across 29 individuals with higher depth sequencing. Different colors represent ROH length categories. The right barplot shows the proportion of genome with ROH (*F*_ROH_ inbreeding coefficient) for each individual with length category colored, ordered by a neighbor-join phylogeny tree based on whole-genomic SNPs. (**B**) Scatter plot of number of ROH (NROH) segments versus sum length of ROH (SROH) for 29 individuals, with Pearson correlation coefficient and *P* value. (**C**) Proportion of genome with ROH across 19 pseudochromosomes. Above the *x* axis shows the wild individual B96_change, and below the *x* axis shows the mean value of the captive population (*n* = 28). The ROH length categories are color coded as in (A). (**D**) Frequency distribution of ROH categories is compared between the wild individual (B96_changle) and the captive population (*n* = 28). (**E**) Percentage of individuals within the captive population that had genomic regions with ROH was presented across pseudochromosomes. (**F**) Inbreeding coefficient estimation for both 29 normal-depth and 215 low-depth sequenced individuals. Founder (1999) is an individual captured from wild field in 1999. ZJ indicates three captive individuals from Zhejiang, China. Wilcoxon test was performed for inbreeding coefficient comparison between 29 normal-depth and 215 low-depth sequenced individuals. ns, non-significant.

Historical demography and recent inbreeding are detectable through runs of homozygosity (ROH) in the genome (*19*, *20*). The number and length of ROHs reflect individual demographic history. Here, we identified ROHs and calculated the ROH-based inbreeding coefficient (*F*_ROH_) for the 29 normal-depth (~18.19×) sequenced individuals. A total of 9705 ROHs that are greater than 1 Mb in length were identified (Fig. 4A and data S2). The sum of ROHs (SROHs) increased with the number of ROHs (NROHs) for all 29 individuals, with the wild individual (sample ID: B96_changle) having the largest SROH and NROH (Fig. 4B and table S16). In addition, the number of ROHs is positively correlated with chromosome length (Pearson correlation *R* = 0.67, *P* value = 3 × 10^−5^) (fig. S6). The average length of ROHs was longest on the LG03 for the captive population and on the LG02 for wild individual (fig. S7). ROHs were further classified into five length categories, with the shortest ROH (1 to 2 Mb) being the most abundant for both wild individual and captive population (Fig. 4, A to D).

The *F*_ROH_ varied from 19.63 to 56.44% across 29 Chinese alligator genomes, with the wild individual (sample ID: B96_changle) having the highest value (56.44%), significantly higher than that of the captive population (*t* test: *P* value < 2.2 × 10^−16^) (Fig. 4, A and B, and table S14). *F*_HOM_ inbreeding coefficient, which measures the heterozygosity reduction, also confirmed that the wild individual B96_changle had the highest inbreeding coefficient (table S15). The average SROH of captive population (*n* = 28) was about 740.23 Mb (SD = 149.70 Mb), accounting for 32.70% (SD = 6.61%) of the genome assembly, which is similar to that of the endangered mountain gorillas (34%) (*21*). The average proportion of genome with ROHs was found to be highest on LG02 for the wild individual and LG05 for the captive population (Fig. 4C). Most genomic regions in ROHs were detected in 37.15% (mean value) individuals of captive population (*n* = 28), while only a few ROH regions present in more than 75% of them (Fig. 4E). The wild individual B96_changle had 31 private ROHs, spanning 3.27 Mb, with a mean length of 105.36 kb (table S16). A total of 80 genes were in these wild individual private ROHs (table S17).

We also calculated the inbreeding coefficient using GL for both 215 low-depth and 29 normal-depth sequenced individuals. The inbreeding coefficient did not significantly differ between different sequencing depths (Wilcoxon text, *P* value = 0.24) (Fig. 4F). The wild founder (sample ID: R99_wild) and three F_1_ generation offspring of captured wild individuals had an estimated inbreeding coefficient of 0 (Fig. 3F). Three captive individuals from the Zhejiang population (ZJ) showed some degree of inbreeding. Two wild individuals (sample ID: B96_changle and R58_CL_wild) captured from Changle, Anhui Province in 2015 exhibited the highest average inbreeding coefficient (Fig. 4F). The increased inbreeding coefficient of captured wild individuals from 1999 to 2015 suggests a potential decline in the wild population. Unexpectedly, captive breeding showed a relatively low average inbreeding coefficient, which could be attributed to the larger breeding populations in the reserve. A few captive individuals exhibited higher levels of inbreeding, possibly due to random mating with little breeding management. The average inbreeding coefficient in the ACANNR was significantly lower than that of the three Zhejiang individuals, possibly due to the larger founder population size in the ACANNR.

These findings support the hypothesis that wild individuals have a higher level of recent inbreeding history than captive individuals due to the smaller population size and habitat shrinkage. The large size of the captive population and greater mating randomness may explain the lower inbreeding level observed in the captive population.

### Demographic history of Chinese alligator

**Fig. 5.**
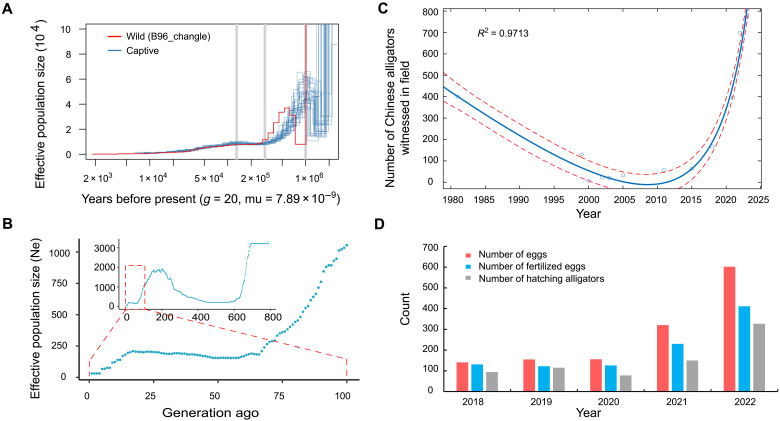
Demographic history inference of Chinese alligator. (**A**) Demographic history inferred from MSMC method using 29 normal-depth sequenced individuals. Generation time of Chinese alligator was set to 20 years, and the mutation rate per generation was set 7.89 × 10^−9^. mu, mutation rate per generation. (**B**) Recent effective population size (Ne) inference 29 normal-depth sequenced individuals using GONe. Ne was averaged more than 40 independent estimates. (**C**) Number of Chinese alligators witnessed in the field. The circles were used to indicate the number of observed wild Chinese alligator in the field. The blue line is the fitted curve. The red dash lines were used to indicate the confidence interval. (**D**) Survey of number of eggs, fertilized eggs, and hatching alligators in the field.

To address the changes in effective population size (Ne) over time, we first estimated historical Ne using the multiple sequentially Markovian coalescent (MSMC) method (*22*). The results showed that the species experienced a great population decline from 1 to 0.3 million years ago, followed by a slow and continuous decline, which aligns with the previous study (*13*) (Fig. 5A). Notably, the demographic histories of captive individuals were similar to each other but differed from that of the wild individual (sample ID: B96_changle). The effective population size is associated with the strength of drift and the probability of inbreeding within the population. In the case of small populations, such as the wild Chinese alligator population, genetic diversity tends to decrease more rapidly than large populations due to stochastic sampling error (i.e., genetic drift).

The recent effective population size of Chinese alligator was estimated using two software, SNeP (*23*) and GONe (*24*), using LD information contained in 29 normal-depth sequenced individuals. GONe revealed a marked decrease in Ne, starting from around 700 generations ago and hitting a low around 580 generations ago. This was followed by a gradual increase, peaking at around 200 generations ago, before experiencing another decline. A period of stability emerged around 66 generations ago, with the Ne hovering near 200, before falling again to approximately 76 at eight generations ago, and ultimately reaching a low of about 31 (Fig. 5B). SNeP also revealed a declining Ne over the past 100 generations, with a drop from 209 individuals around 98 generations ago to just 73 at 13 generations past (fig. S8). Both SNeP and GONe consistently point to a decreasing trend and a small recent Ne, which is in agreement with the well-documented history of alligator’s recent near extinction due to poaching and loss of habitat.

From 1981 to 2023, the Chinese alligator population experienced notable fluctuations. In 1981, a joint Sino-American survey conducted by M. E. Watanabe from the United States and Anhui Normal University estimated about 300 to 500 existing Chinese alligators, predominantly juveniles or subadults (*25*). In 1999, the Wildlife Conservation Society and East China Normal University reported fewer than 130 individuals in the wild, with an estimated decline rate of 6 to 8% per year (*26*). Subsequently, Ding *et al.* (*27*) reported fewer than 120 wild Chinese alligators (including those in Zhejiang) and estimated the population was still declining at a rate of 4 to 6%. However, since 2015, annual surveys of the wild Chinese alligator population have shown a year-on-year increase, indicating great success in the conservation efforts for this species (Fig. 5C). Moreover, between 2018 and 2022, our annual surveys on the breeding status of wild Chinese alligators in China revealed an increasing trend in nesting, egg laying, and hatching alligator numbers, with particularly notable growth in the past 2 years (Fig. 5D). Now, the observed wild population of the Chinese alligator has reached 773 individuals (Fig. 5C). Overall, following declines, the Chinese alligator population has shown remarkable recovery due to effective conservation measures and reintroduction programs. This reveals the success of the Chinese alligator conservation program while also highlighting areas that still need attention and improvement, such as breeding success rates and long-term population stability.

## DISCUSSION

### Genetic diversity of wild and captive population

Genetic diversity is intrinsically linked to species responses to environmental change, making it a key factor in drafting conservation management for threatened species (*3*, *4*, *28*, *29*). In populations or species experiencing population bottlenecks, the degree of inbreeding and genetic drift increases, which further leads to the accumulation or even fixation of deleterious mutations (*2*, *5*, *29*). This study reveals that the Chinese alligator population has low level of genetic diversity by calculating the genome-wide heterozygosity (fig. S9 and table S13). Previous genetic study based on microsatellite data also indicated low genetic diversity (*30*). This finding aligns with historical records of Chinese alligator occupancy across the relative narrowly of habitats and the small recent effective population size. Certain alligator populations reveal signatures of recent inbreeding and founder events or indicate population bottlenecks and isolation.

A detailed examination of landscapes and habitats highlights notable differences: The wild population is dominated by variable habitats amid a matrix of extremely high human population densities, whereas for the captive populations, human density and human disturbance are low, and habitat with high quality is more continuous. We estimated that, in contrast to the mere ~200 adult wild individuals, the captive population may consist of 15,000 individuals.

We propose that extreme fragmentation and high human population density in the wild population of Chinese alligators have resulted in isolated populations, where individuals may be more likely to mate with their relatives. Persistent human impacts, such as fragmentation, likely disrupt natural evolutionary processes in wild populations. Our findings from genetic diversity, population structure analyses, and demographic history reaffirm their unique management status, which suggests that the management status of the wild alligator population should be re-evaluated given their antiquity and potential genetic distinctiveness.

Valuably, the relatively higher heterozygosity and lower inbreeding level in the captive population compared to the wild population bode well for their future survival and population recovery. It is critical for future conservation efforts to prioritize wild population recovery, gene flow through connectivity, and the promotion of increases in the size of the wild population. Restoring and maintaining gene flow between populations through habitat corridors is crucial, alongside increasing population size. Assisted gene flow could be considered as a management strategy, particularly when inbreeding is associated with the loss of fitness and potentially inbreeding depression.

### Inbreeding history of Chinese alligator

Critically endangered species often suffer from population decline problems such as loss of genetic diversity, increased inbreeding, and genetic drift, resulting in elevated genetic load and reduced adaptability and viability (*2*, *4*, *5*). Inbreeding usually leads to decreases in the vigor and reproductive fitness of offspring, which is named inbreeding depression. Increased inbreeding exposes lethal recessive and deleterious partial recessive mutations, while purifying selection can reduce the frequency of deleterious mutations (*31*, *32*). However, in extremely small populations, the effectiveness of purifying selection will be substantially weakened. In the protection and management of endangered species, it is particularly important to evaluate their genetic background such as inbreeding and relatedness.

Longer ROH corresponds to more recent inbreeding events (mating between closely related individuals), while shorter ROH corresponds to more ancestral inbreeding events. The number of ROHs in the wild individual (sample ID: B96_changle) is significantly larger than the average value of captive population across four longer ROH length categories (1 to 2, 2 to 4, 4 to 8, and 8 to 16 Mb), suggesting that the wild population may have suffered more inbreeding recently due to the population decline and habitat shrinkage (Fig. 4D). The calculation of inbreeding coefficient of whole 244 individuals confirmed this conclusion with two additional wild individuals (sample IDs: R58_CL_wild and R99_wild).

The identification of ROH may be influenced by parameters, population-specific demographic history, which sometimes renders it not incomparable across different studies (*33*). Notably, the recent study made by Yang *et al.* (*15*) reported the wild founder of CX population, CX1, had the lowest *F*_ROH,_ which was 46.72%. We also noticed that CX1 had fewest longest ROH (>10 Mb) among all individuals, implying a minimal occurrence of recent inbreeding events in the CX1 lineage, potentially explaining its low *F*_ROH_ value. Conversely, our analysis of the wild individual, B96_changle, demonstrated a significantly larger number of longer ROHs (4 to 8 and 8 to 16 Mb) than the average of captive population and the highest *F*_ROH_ among 29 individuals, indicating a higher occurrence of recent inbreeding events in this wild lineage (Fig. 4, A and D). Similarly, another wild individual (sample ID: R58_CL_wild), which was also captured in Changle in 2015 and identified as having first-degree relationship with B96_changle, also showed high inbreeding coefficient as B96_changle (Fig. 4F).

The contrast observation of *F*_ROH_ for wild individuals B96_changle, and CX1 is particularly concerning because B96_changle should probably be younger than CX1, suggesting that the wild population in Changle could be under increasing environmental and anthropogenic pressure. While the sampling locations of two individuals are different (Changxing, Zhejiang versus Changle, Anhui), which means that the demographic history of these two individuals could be different.

The 28 captive individuals sampled from protected areas (ACANNR), that harbor large and connected populations, displayed lower ROH based inbreeding coefficient and shorter ROHs compared to the wild individual, B96_changle. Moreover, the captive population exhibited higher heterozygosity compared to the wild population. These findings are highly encouraging for the ongoing efforts to restore the alligator population. This positive trend can be attributed largely to the effective conservation measures implemented by ACANNR, leading to a stable current population.

### Relatedness inference for strict breeding management

Given the potential risks associated with inbreeding, strict breeding management practices are essential for maintaining genetic diversity and minimizing the negative effects of inbreeding depression. This includes careful selection of breeding pairs based on genetic compatibility and kinship analysis. In reintroduction programs, the release of captive-bred individuals into the wild fields aims to supplement dwindling wild populations. However, it is crucial to prevent the release of individuals with close kinship ties into the same reintroduction site. Advanced genetic techniques, such as relatedness inference, have facilitated the identification of close kinship relationships within captive populations.

In this study, we compared the performance of relatedness inference by different software and sequencing depth. We observed the different relatedness inferences in the Chinese alligator when using different software or under different sequencing depths similar to as what is reported in other studies (*17*, *18*). However, there is still a strong collinearity between the estimated results by different software or under different sequencing depth with same software, which can still meet our breeding management requirements of selecting extremely unrelated pairs for breeding.

### Limitation and future research

This study has a limitation that warrants acknowledgment: The limited number of wild individuals included in the analysis reduces the comprehensiveness of our assessment of the genetic diversity and structure of wild populations. Consequently, this constraint may lead to an underestimation of the genetic status of these populations. The lack of phenotypic characterization data for Chinese alligators hampers our ability to establish robust phenotype-genotype associations. Addressing this gap through future research would significantly enhance our understanding of the evolutionary dynamics, adaptive potential, and conservation needs of Chinese alligators.

## MATERIALS AND METHODS

### Sample collection

For de novo genome assembly, a female subadult Chinese alligator (approximately 4 years old, measuring 0.86 m in length and weighing 2.35 kg; sample ID: 20191201) was selected from ACANNR in 2019. For population genomic analysis, 29 individuals were selected for normal-depth (~18.19×) next-generation sequencing in 2015. This cohort included one wild individual (sample ID: B96_changle; umbilical tissue) captured from wild field in Changle, Anhui, China and 28 captive individuals from ACANNR. In addition, 215 individuals from three sources were sampled for low-depth (<5×) sequencing. Among these 215 individuals, three captive individuals (sample IDs: R93_ZJ, R94_ZJ, and R95_ZJ; blood samples) were collected from ZJ, China in 2015; three captive individuals (sample IDs: R94_F1, R97_F1, and R98_F1; blood samples), representing the F_1_ generation of the captured wild population were collected in ACANNR in 1999; one wild individual (sample ID: R58_CL_wild; umbilical tissue) was captured from Changle, Anhui Province, China in 2015; and one wild individual (sample ID: R99_wild; blood sample) was captured in the field (exact location unknown) in 1999 for the original construction of the artificial breeding population in the nature reserve. The remaining 207 individuals were collected from captive populations in ACANNR. Detailed sample information is shown in table S12. All samples (scales or blood) were collected by noninvasive sampling during the individual medical examination and artificial breeding population construction, and all procedures were approved by the Animal Care and Welfare Committee of Anhui Normal University (approval number: 2020005).

### DNA and RNA extraction, library construction, and sequencing

For de novo genome assembly, the total genomic DNA was extracted from fresh liver tissue using the phenol/chloroform extraction method. The extracted genomic DNA was subsequently sequenced on the Oxford Nanopore PromethION platform (table S2). Blood sample from same individual was used for Hi-C library construction. The blood sample (150 μl) was collected using PAXgene Blood DNA Tubes (QIAGEN) and cross-linked with formaldehyde (final concentration of 1%) for 10 min, and then glycine (final concentration of 0.2 M) was added for 5 min to terminate the cross-linking process. Following (*34*), Hi-C libraries were constructed with insert size ranging from 300 to 700 base pairs (bp) and subsequently sequenced on the Illumina NovaSeq 6000 platform. Approximately 116.03-Gb clean Hi-C data was generated. For genome survey and genome assembly polishing, three DNA libraries with insertion lengths of about 350 bp were constructed and sequenced on the Illumina platform with paired-end 150 bp in length, generating a total of 213.32-Gb data. For genome annotation, the total RNA from 32 individuals were extracted using TRIzol reagent and sequenced with Illumina NovaSeq 6000 platform with paired-end 150 bp in length. For the population genomics analysis, 29 and 215 individuals were sequenced on the Illumina platform with normal-depth and low-depth sequencing, respectively.

### Genome assembly with nanopore long reads, Illumina reads and Hi-C data.

Nanopore long reads were self-corrected using Canu (*35*). The corrected reads were then used for de novo assembly using wtdbg2 (https://github.com/ruanjue/wtdbg). To improve the quality of our genome assembly, we performed three rounds of polishing with the Nanopore long reads using Racon (*36*) and three rounds of polishing with Illumina data using Pilon (*37*). To improve the continuity of the assembly (782 contigs), about Hi-C data were (116.03 Gb) used to perform scaffolding. Raw data were first filtered using Hic-Pro (v2.10.0) (*38*). Before Hi-C scaffolding, we first performed a preassembly error correction of scaffolds, which required the splitting of scaffolds into segments of average length of 50 kb. Hi-C data were mapped to these segments using Burrow-Wheeler Aligner (BWA) (version 0.7.10-r789) (*39*), and uniquely mapped reads were retained to perform the assembly by using LACHESIS (*40*). Any two segments that showed inconsistent connection with information from the raw scaffold were checked manually. These corrected scaffolds were then assembled with LACHESIS (parameters for running LACHESIS included: CLUSTER_MIN_RE_SITES, 136; CLUSTER_MAX_LINK_DENSITY, 2; CLUSTER_NONINFORMATIVE_RATIO, 2; ORDER_MIN_N_RES_IN_TRUN, 15; ORDER_MIN_N_RES_IN_SHREDS, 15). After this step, group clustering, placement, and orientation errors exhibiting obvious discrete chromatin interaction patterns were manually adjusted. A total of 95.04% of unique mapped read pairs were valid interaction pairs and used for correction of scaffolds and clustered, ordered, and orientated contigs into chromosomes by LACHESIS (table S3).

### Genome assembly evaluation

The completeness of assembly was assessed using BUSCO (version 3.1.0) (*41*) with the tetarpoda_odb9 lineage dataset (creation date: 13 February 2016; 3950 BUSCOs). CEGMA v.2.5 (*14*) database (458 conserved core genes in eukaryotes) was also used to assess the completeness of the genome assembly with default parameters sets. For estimating the alignment rate, clean DNA-seq and RNA-seq reads were aligned to the assembly using BWA (v0.7.12) (*39*) and Hierarchical Indexing for Spliced Alignment of Transcripts (HISAT) (version 2.0.4) (*42*), respectively. In addition, we performed pairwise genome sequence comparisons for current chromosome-scale assembly (reference) and previously released scaffold-level assembly (query) (*13*) using MUMmer (version 4.0.0) (*43*). SNPs between current assembly and previous assembly were detected using a couple of the MUMmer components. The program nucmer was used to align query sequence to reference sequence. Then program delta-fliter was used to filter the alignments, selecting only these alignments that comprise the one-to-one mapping between reference and query. Last, program show-snps was used to report the SNPs and small InDels with parameters -Clr.

### Repeats identification and genome annotation

We used LTR_DINDER (*44*) and RepeatScout (*45*) to construct a repeat sequence database for the assembly. PASTEClassifier (*46*) was used to classify the repetitive sequence in the database. The species-specific repeat library was combined with Repbase as the final repeat sequence database, and then the RepeatMasker (*47*) was used to predict the repeat sequences in genome using the constructed repeat sequence database. LTR_FINDER, RepeatScout, and PASTEClassifier were run with default parameters; RepeatMasker parameters were modified as following: -nolow -no_is -norna -engine wublast.

Three strategies were used in gene prediction: ab initio prediction, prediction based on homologous species, and prediction based on the transcriptome. Ab initio prediction was performed using GENSCAN (*48*), Augustus (version 2.4) (*49*), GlimmerHMM (version 3.0.4) (*50*), GeneID (version 1.4) (*51*), and SNAP (version 2006-07-28) (*52*). GeMoMa v1.3.1 (*53*, *54*) was used for homology prediction with five related species (*Danio rerio*, *Alligator mississippiensis*, *A. sinensis*, *Crocodylus porosus*, and *Gavialis gangeticus*). HISAT (version 2.0.4) (*42*) and Stringtie (version 1.2.3) (*55*) were used for both genome-guide and de novo transcript assembly. TransDecoder v2.0 (https://github.com/TransDecoder/TransDecoder) was used to identify coding regions within transcripts. GeneMarkS-T (version 5.1) (*56*) was also used for ab initio prediction of protein-coding regions in transcripts. PASA (version 2.0.2) (*57*) was used to generate a comprehensive transcript database using the trabnscript assemblies by both genome-guided and de novo RNA-seq. Last, EVM (version 1.1.1) (*58*) was used to integrate the prediction results obtained by the above three methods (*57*).

For gene function annotation, we aligned all protein-coding genes to protein databases, including Non-Redundant database of protein sequences (NR) (*59*), EuKaryotic Orthologous Groups (KOG) (*60*), Gene Ontology (GO) (*61*), Kyoto Encyclopedia of Genes and Genomes (KEGG) (*62*), and TrEmbl (*63*) using Basic Local Alignment Search Tool (BLAST) (−*e* value, 1 × 10^−5^).

Rfam database (*64*) was used to identify microRNA, rRNA, and other noncoding RNA families by using Infernal (version 1.1.4) (*65*). tRNAscan-SE (version 2.0) (*66*) was used to identify tRNA genes. For pseudogene prediction, GeneWise (*67*) was used to find immature stop codons and frameshift mutation in the gene sequence.

### Read processing, variant calling, and SIFT annotation

Raw reads were filtered, and low-quality bases were trimmed by fastp (version 0.23.2) with default parameters (*68*). Bowtie2 (version 2.2.5) was used to map the clean reads onto the reference genome (*69*). SAMtools (version 1.15) was then used to sort and index these alignment files (*70*). Picard (version 2.26.10) (https://github.com/broadinstitute/picard) was used to remove duplicates within the alignment files with parameters “-AS true -REMOVE_DUPLICATES true”. For variant calling, we used the Genome Analysis Toolkit (GATK, version 4.2.2.0) haplotypeCaller module with Genomic VCF (GVCF) mode (*71*). The CombineGVCFs module within GATK was then used to combine all gvcf files of a specific subset. The GenotypeGVCFs module within GATK was used to transform the combined gvcf file into VCF format. SNPs and InDels were separate using module SelectVariants within GATK from the VCF file. SNP filtering was performed by module VariantFiltration within GATK (parameters: -window 10 -cluster 3 -filter QD < 2.0 -filter QUAL <30.0 -filter MQ < 40.0 -filter FS > 60.0 -filter ReadPosRankSum < −8.0 -filter MQRankSum < −12.5 -filter SOR > 3.0). InDel filtering was conducted by module within GATK (parameters -filter QD < 2.0 -filter QUAL <30.0 -filter FS > 200.0 -filter ReadPosRankSum < −20.0). For genotype imputation of 215 low-depth sequenced individuals, we first integrated the GVCF files of 29 normal-depth sequenced individuals with 215 low-depth sequenced individuals and then filtered these SNP sites with a missing rate > 0.8 and an MAF < 0.01. A total of 402,316 SNP sites were retained for imputation using Beagle (version 5.4) (*72*). The predicted protein sequences of Chinese alligator generated in current study and UniProt database (release 2024–03) were used to build our own genomic database with SIFT prediction with script make-SIFT-db-all.pl provided by SIFT4G_Create_Genomic_DB (*73*). SIFT4G_Annotator was then employed to annotate the VCF file with our own genomic database.

### SV analysis

Delly (v 1.2.6) was used to genotype the SVs using the paired-end reads from 29 individuals sequenced with normal depth (*74*). The clean reads were first mapped against the reference genome with bowtie2 (*69*). Then, the alignment files were sorted by coordinate, and duplicates were removed using SAMtools and Picard, respectively (*70*). Delly merge was used to merge SV sites of 29 samples into a unified site list. Regenotyping for the merged SV site list was conducted across all 29 samples using DELLY call. The final SVs were then used to perform PCA using R package FactoMineR. Then, the maximum likelihood phylogeny tree was reconstructed using IQ-TREE (version 2.0.7) with 1000 bootstrap replicates (*75*).

### Population structure analyses

For 29 normal-depth sequenced individuals, PLINK (version 1.9) was used to perform PCA using the whole genomic SNPs. In addition, R package SNPRelate (version 3.17) (*76*) was used to perform PCA for both 29 normal-depth sequenced and the combination of 29 normal-depth and 215 low-depth sequenced individuals. SNPs with MAF < 0.01 were filtered. Then, LD-pruned SNPs were generated by LD threshold < 0.5 for PCA by SNPRelate. Maximum likelihood tree based on whole genomic SNPs for 29 normal-depth sequenced individuals was reconstructed using IQ-TREE (version 1.6.12) with 1000 bootstrap replicates (*75*). The resulting tree was visualized using FigTree (version 1.4.4) (https://github.com/rambaut/figtree).

### Relatedness inference and inbreeding coefficient estimation

For relatedness inference, four different software, KING (version 2.2.7) (*77*), VCFtools (version 0.1.17) (*78*), SNPRelate (version 3.17) (*76*), and NgsRelate (*16*) were used. KING was used with parameter –kinship on 1.3 M genome-wide SNPs for 29 normal-depth sequenced individuals. VCFtools was used with relatedness2 option. For the relatedness inference of both 29 normal-depth and 215 low-depth sequenced individuals, we assigned relationship degree to each pair of individuals by using the KING-robust kinship coefficient generated by NgsRelate. The inference criteria for duplicates/Monozygotic (MZ) twins, first-degree, second-degree, third-degree, and unrelated relationships are as follows: >$\frac{1}{2^{3/2}}$, ($\frac{1}{2^{5/2}}$, $\frac{1}{2^{3/2}}$), ($\frac{1}{2^{7/2}}$, $\frac{1}{2^{5/2}}$), ($\frac{1}{2^{9/2}}$, $\frac{1}{2^{7/2}}$), and <$\frac{1}{2^{9/2}}$. Inbreeding coefficients for both 215 low-depth and 29 normal-depth sequenced individuals were estimated by NgsRelate using GLs instead of called genotypes. The GL file of 244 individuals was generated by angsd (parameters: -gl 2 -domajorminor 1 -snp_pval 1e-6 -domaf 1 -minmaf 0.05 -doGlf 3). In addition, we used PLINK (version 1.9) (*79*) to calculate inbreeding coefficients for 29 normal-depth sequenced individuals using genome-wide SNPs with parameters --het.

### Genomic heterozygosity

The genomic heterozygosity, or genetic variability of 29 normal-depth sequenced individual was calculated as the number of SNP sites in the heterozygous state divided by the total length of reference genome. For these 215 low-depth sequenced individuals, we calculated the proportion of heterozygous SNPs by calculating the number of SNP sites in the heterozygous state divided by total number of SNP sites for both genotype results before and after genotype imputation.

### ROH and inbreeding coefficient

To investigate more recent demographic history and the level of inbreeding of captive and wild populations of Chinese alligator, we estimated inbreeding by identifying ROH with the sliding-window approach implemented in PLINK (version 1.9) (*79*). Specifically, the parameters used were: -homozyg-density 50 -homozyg-gap 1000 -homozyg-kb 1000 -homozyg-snp 100 -homozyg-window-het 1 -homozyg-window-snp 50 -homozyg-window-threshold 0.05. The autozygous runs obtained were classified into various lengths (runs between 1 and 1, 2 and 4, 4 and 8, and 8 and 16 Mb, as well as runs longer than 16 Mb). The inbreeding coefficients based on ROH (*F*_ROH_) was estimated by calculating the total length of the genome in ROH longer than 1 Mb and dividing by the total length of our genome assembly.

### Demographic history

We used MSMC method to infer the demographic history of the 29 normal-depth sequenced individuals (28 captive and 1 wild). The method reconstructs the history of change in effective population size over time using the distribution of the most recent common ancestor between two alleles within an individual. As MSMC inference does not have enough power for recent datings due to limited recombination events in a short time, we used GONe (version 1.03) and SNeP (version 1.1) (*23*, *24*), which both suited for estimating recent effective population size in single closed populations based on the pairwise LD between markers.

### Annual surveys of the wild Chinese alligator population

Three different methods were employed in the field survey of wild Chinese alligator. Nighttime light Counting Method is the primary survey method. This method counts Chinese alligators by utilizing the characteristic reflection of orange-red light from their eyes under flashlight illumination from dusk to early morning (about 18:00 p.m. to next day 6:00 a.m.). During this period, surveyors walk slowly along the land surrounding the water body using flashlights to scan the entire water surface, paying special attention to water edges concealed by vegetation. They record the number of Chinese alligators observed. Then, the flashlight is turned off, and they wait silently for 5 min before repeating the observation at least three times. The number of alligators observed is recorded, along with an estimate of their body size. At least two different points are chosen at each pond. For large reservoirs, surveyors travel on inflatable rafts to observe as much of the area as possible. Key survey locations with poor results due to weather or other reasons can be revisited for additional surveys. The following two supplementary survey methods were also used. (i) Call playback method: This method uses the sound of alligator bellowing to confirm their presence. It involves using an MP3 player to play the authentic distress calls of juvenile alligators to lure adult alligators for observation. (ii) Daytime trace survey: This involves understanding the distribution traces of Chinese alligators (which can be combined with interview surveys). The main focus is to search for their burrows and nesting sites along the banks of ditches, ponds, reservoirs, and other bodies of water in the distribution area. This is done by investigating imprints such as feces, footprints, and tail marks to further confirm the presence of alligators. This also helps in planning routes for nighttime surveys and studying their surrounding habitats.

## Supplementary Materials

### The PDF file includes:

Figs. S1 to S9 Tables S1 to S18 Legends for data S1 and S2 References
