# Supplementary material for: Genomic insights and the conservation potential of captive breeding: The case of Chinese alligator
Source: Sci Adv. 2025 Apr 2;11(14):eadm7980. doi: 10.1126/sciadv.adm7980 (PMC11963981; doi:10.1126/sciadv.adm7980)
Supplement: Supplementary file 1 — Figs. S1 to S9 Tables S1 to S18 Legends for data S1 and S2 References [file sciadv.adm7980_sm.pdf]

Science Advances
AAAS

Supplementary Materials for

Genomic insights and the conservation potential of captive breeding: The case of Chinese alligator

Tao Pan *et al.*

Corresponding author: Xiaobing Wu, wuxb@ahnu.edu.cn.

Sci. Adv. **11**, eadm7980 (2025)
DOI: 10.1126/sciadv.adm7980

The PDF file includes:

Figs. S1 to S9
Tables S1 to S18
Legends for data S1 and S2
References

Other Supplementary Material for this manuscript includes the following:

Data S1 and S2

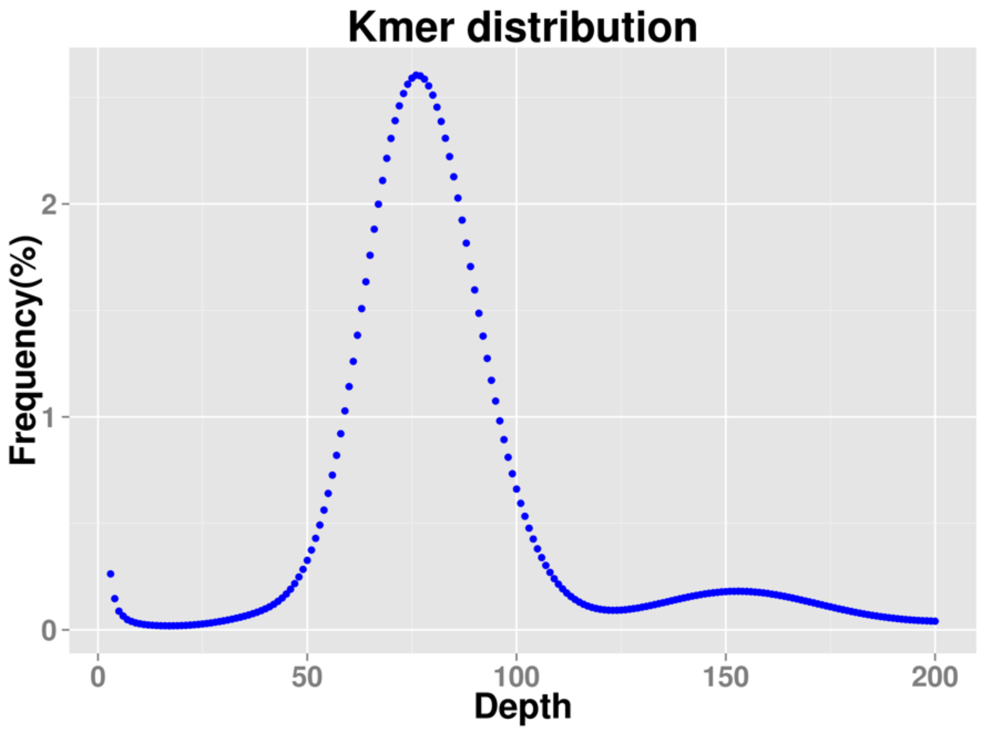

Fig. S1. Genome survey based on 17-mer frequency distribution.
Illumina short reads were employed to estimate the genomic characteristic of Chinese alligator.

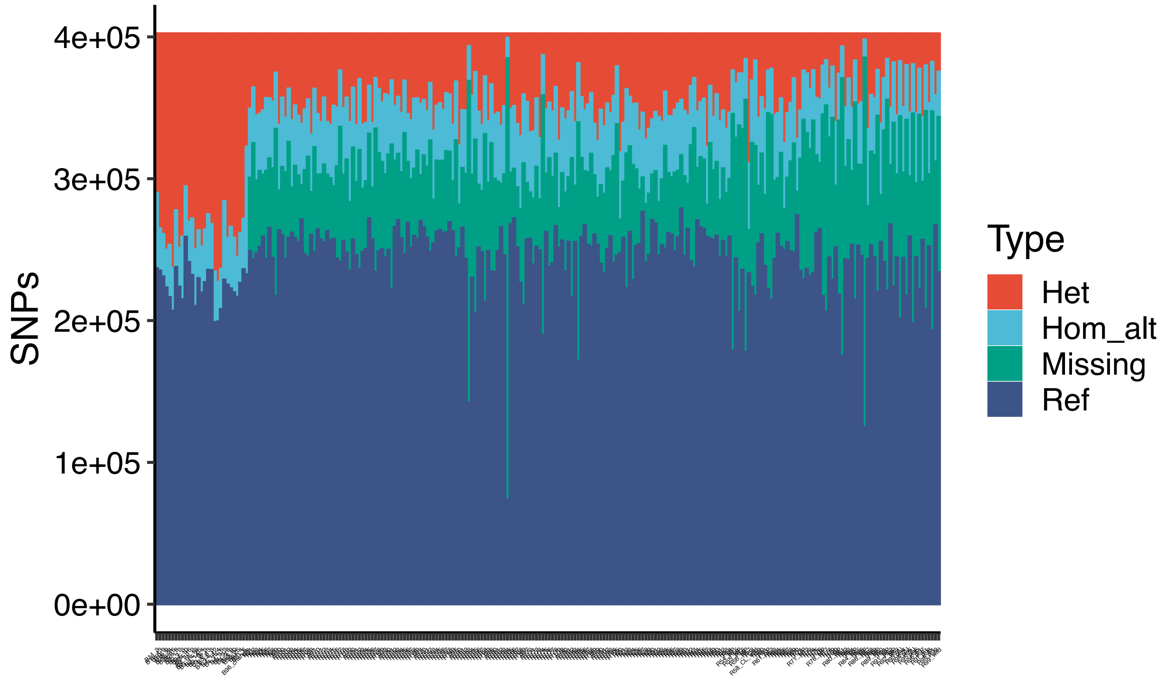

Fig. S2. SNP genotype statistic summary for 29 individuals sequenced at normal depth and 215 individuals sequenced at low depth.

Het is short for heterozygous SNPs. Hom_alt is short for homozygous alternative genotype SNPs. Ref is short for homozygous reference genotype SNPs.

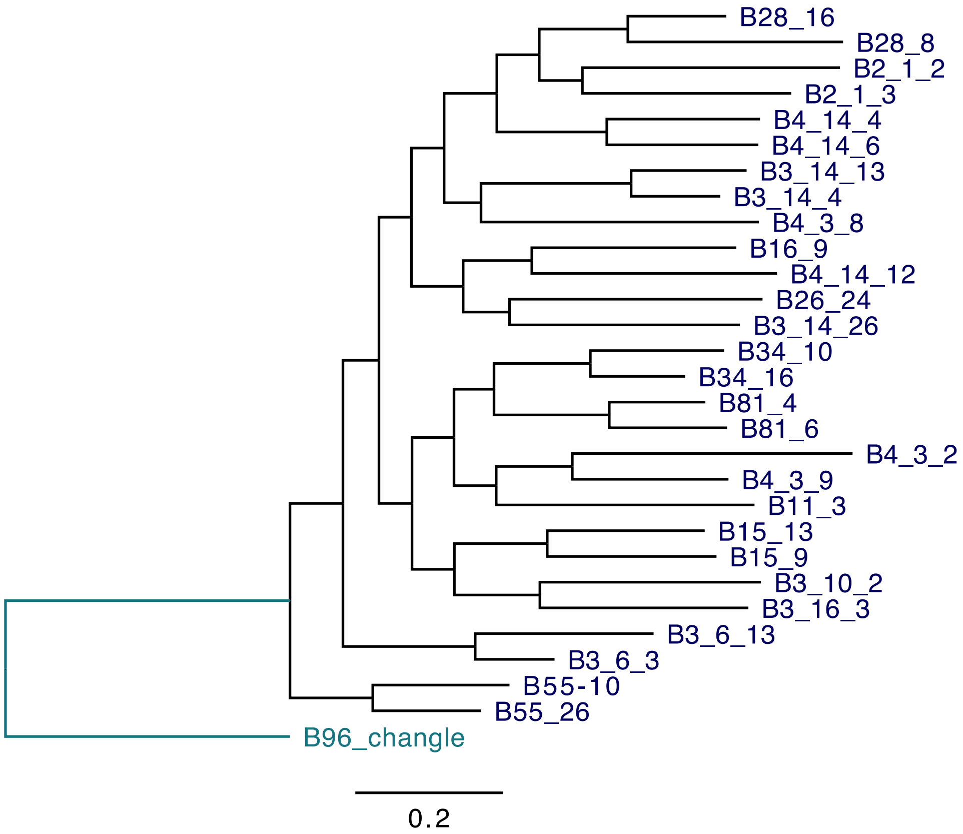

Fig. S3. Maximum-likelihood phylogeny tree reconstructed using whole genomic SNPs of 29 individuals sequenced at normal depth.

Bootstrap support values below 100 are shown. The following samples have recorded as sampled from same clutches: (B28_16 and B28_8), (B2_1_2 and B2_1_3), (B4_14_4, B4_14_6 and B4_14_12), (B3_14_4, B3_14_13 and B3_14_26), (B34_10 and B34_16), (B81_4 and B81_6), (B4_3_2 and B4_3_9), (B15_9 and B15_13), (B3_6_3 and B3_6_13), (B55_10 and B55_26).

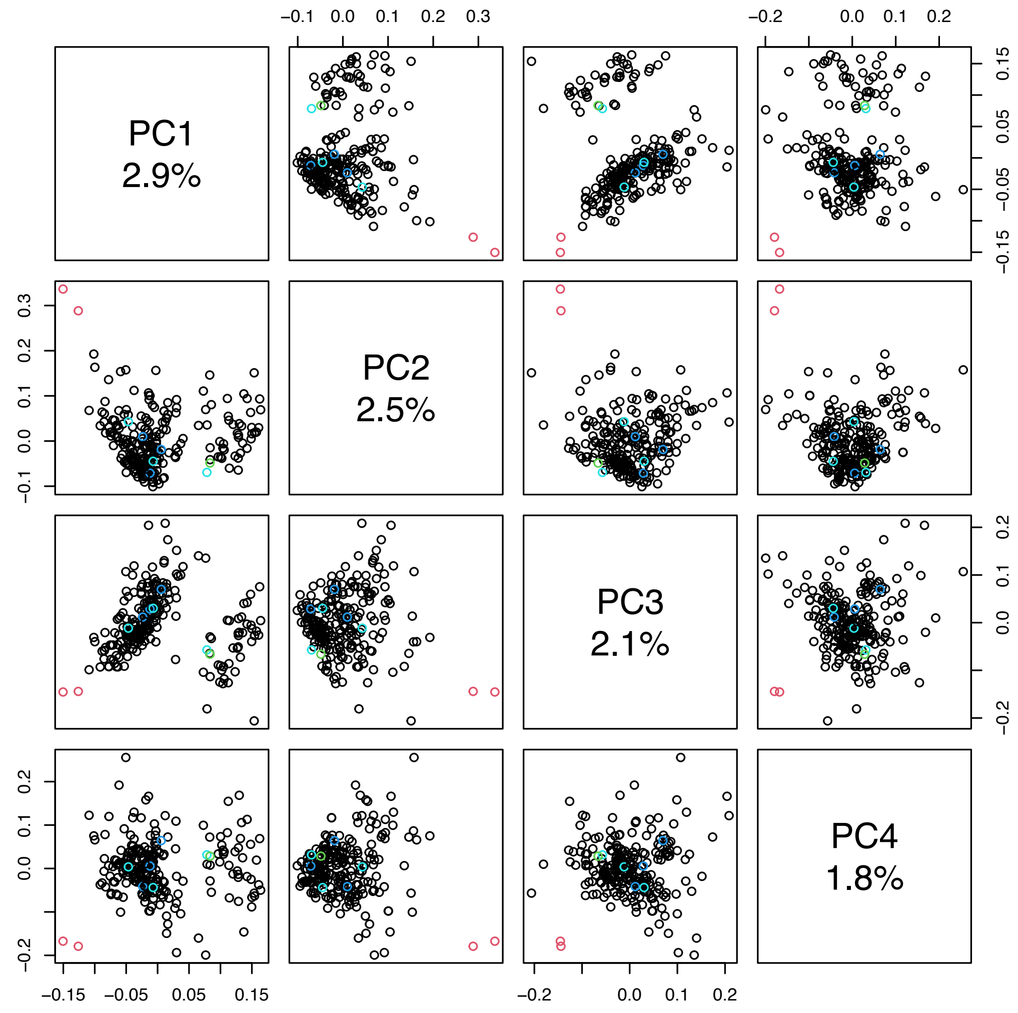

Fig. S4. Principle component analysis for 215 individuals.
A total of 53,972 LD-pruned SNPs were used to perform the analysis. The LD threshold was set as 0.5. The red circles were used to indicate two wild individuals (sample ID: B96_changle and R58_CL_wild), both were captured in Changle, Anhui Province, China in 2015. The green circle was used to indicate the wild individual (sample ID: R99_wild) that captured in 1999 for the original population construction in ACCNNR. The cyan circles were used to indicate the F1 generation individuals of captured wild population. The light blue circles were used to indicate the three individuals from Zhejiang Province (ZJ).

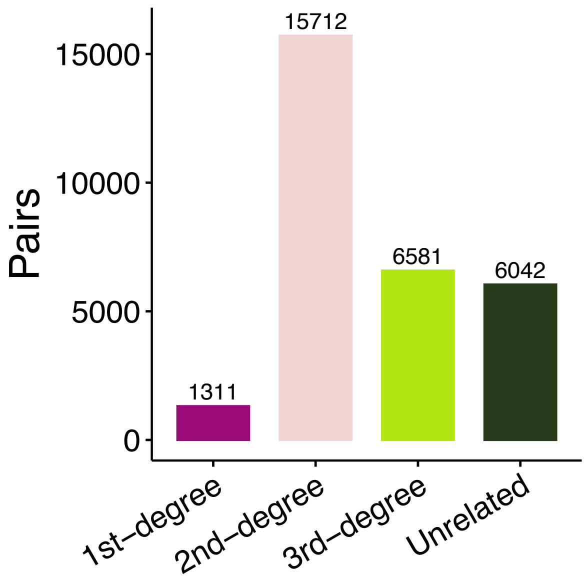

Fig. S5. The number of each relationship degree pairs.
KING-robust kinship coefficient were calculated using NgsRelate for 215 individuals. We assigned relationship degree to each pair using the KING-robust kinship coefficient. The inference criteria for duplicates / MZ twins, first-degree, second-degree, third-degree and unrelated relationships are as follows: > $\frac{1}{2^{3/2}}$, $(\frac{1}{2^{5/2}}, \frac{1}{2^{3/2}})$, $(\frac{1}{2^{7/2}}, \frac{1}{2^{5/2}})$, $(\frac{1}{2^{9/2}}, \frac{1}{2^{7/2}})$, and < $\frac{1}{2^{9/2}}$.

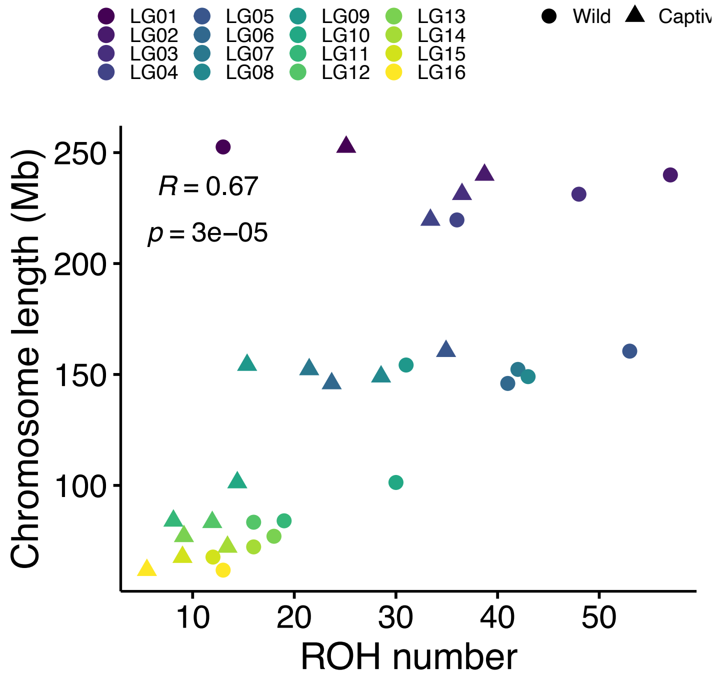

Fig. S6. The correlations between number of ROHs on each chromosome and chromosome length (Mb).

Pearson correlation coefficient was calculated (R = 0.67, p value = 3e-05). The triangles were used to represent the mean number of ROH of each chromosome of captive population. The points were used to represent the mean number of ROH of each chromosome of wild individual (sample ID: B96_changle).

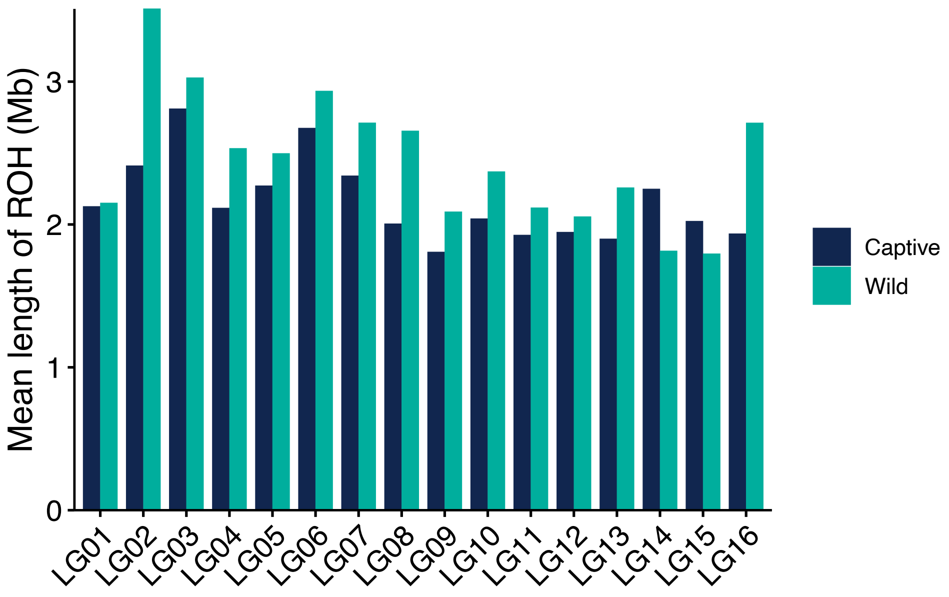

Fig. S7. The mean length of ROH for captive and wild population.
A total of 28 captive-bred individuals and one wild individual, which sequenced with normal-depth, were included in the analysis.

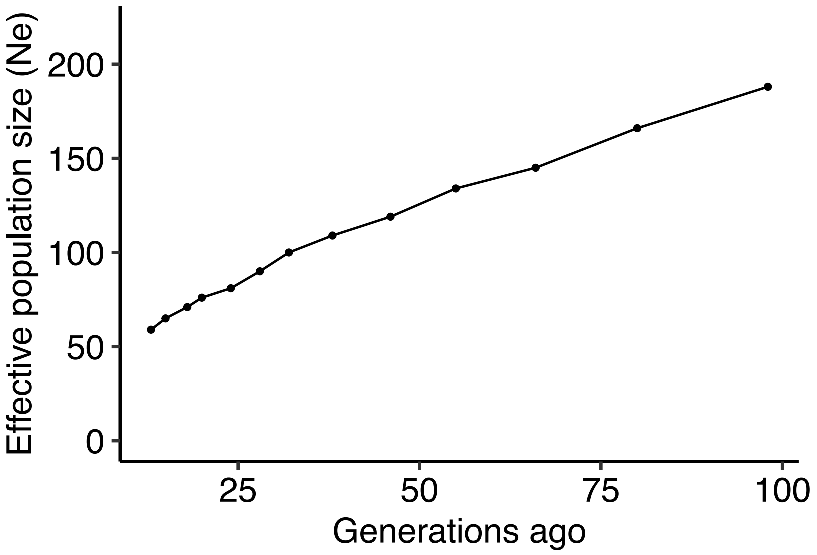

Fig S8. Recent effective population size (Ne) inference based on 29 normal-depth sequenced individuals using SNeP.

The inference was conducted using the 29 normal-depth sequenced individuals.

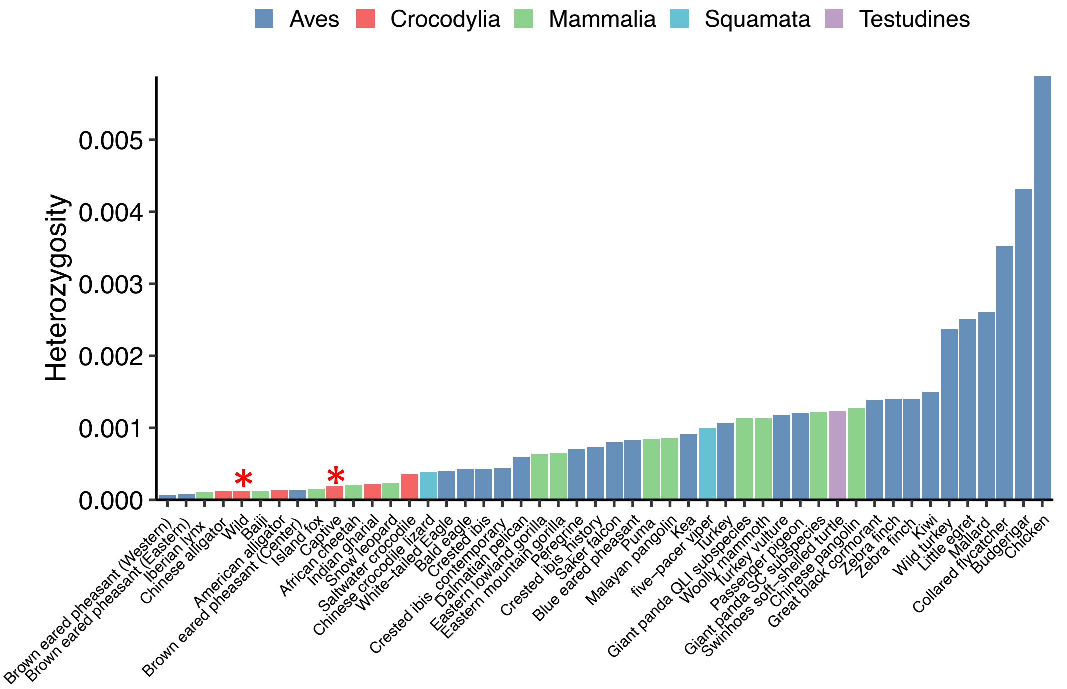

Fig. S9. Comparison of genome-wide heterozygosity among Aves, Crocodylia, Mammalia, Squamata and Testudines.

The red * was used to indicate the average heterozygosity of wild Chinese alligator (sample ID: B96_changle) and the captive individuals (n = 28) in current study. The references of data sources were listed in Table S18 and related literatures were listed in reference section here.

Table S1. Statistical summary for Oxford Nanopore sequencing dataset.

SeqNum	SumBase	N50Len	N90Len	MeanLen	MaxLen	MeanQual
11,040,220	230,946,760,105	25,921	12,146	20,918	253,098	9

SeqNum: number of Oxford Nanopore clean sequence; SumBase: sum of bases; N50Len: N50 sequence length. Its value represents the length of the shortest read in the group of longest sequences that together represent (at least) 50% of the nucleotides in the set of sequences.; N90Len: N90 sequence length; MeanLen: mean length of this sequence dataset; MaxLen: maximum length in this sequence dataset; MeanQual: mean quality of sequences in this dataset.

Table S2. Read length distribution summary for Oxford Nanopore Sequencing dataset.

Length	Reads num	Total length	Percent	Mean length
2000~5000	590,936	2,085,457,511	0.90%	3,529
5000~10000	1,172,111	8,939,716,787	3.87%	7,627
10000~20000	4,815,477	70,220,484,795	30.40%	14,582
20000~30000	2,265,136	55,460,587,353	24.01%	24,484
30000~40000	1,132,654	38,950,304,603	16.86%	34,389
40000~50000	571,596	25,418,559,171	11.00%	44,469
50000~60000	291,400	15,845,784,116	6.86%	54,378
60000~70000	127,861	8,214,110,014	3.55%	64,242
70000~80000	47,932	3,553,800,566	1.53%	74,143
>=80000	25,117	2,257,955,189	0.97%	89,897

Table S3. Statistical summary for Hi-C scaffolding.

Group	Cluster Num	Cluster Length (bp)	Order Num	Order Length (bp)
LG01	40	252,864,459	35	252,539,428
LG02	14	240,127,909	13	239,931,775
LG03	84	231,794,526	72	231,245,342
LG04	18	219,685,974	17	219,648,541
LG05	10	160,514,088	10	160,514,088
LG06	32	146,448,076	27	145,957,654
LG07	14	152,320,531	13	152,285,832
LG08	8	149,054,752	7	149,025,459
LG09	47	154,495,874	41	154,255,617
LG10	4	101,328,041	4	101,328,041
LG11	92	84,245,137	88	84,037,248
LG12	14	83,495,923	12	83,409,468
LG13	20	77,543,710	17	77,074,114
LG14	7	72,323,795	6	72,286,667
LG15	4	67,887,800	3	67,702,714
LG16	156	64,413,949	125	61,802,454
Total (%)	564 (72.12)	2,258,544,544 (99.78)	490 (86.88)	2,253,044,442 (99.76)

Table S4. Mapping summary of Hi-C dataset

Type	Number	Ratio (%)
Total Read Pairs	387,369,017	100
Mapped Reads	747,669,660	96.51
Unique Mapped Read Pairs	308,863,188	79.73
Valid Interaction Pairs	293,547,217	95.04
Dangling End Pairs	5,387,353	1.74
Re-ligation Pairs	4,421,661	1.43
Self-cycle Pairs	552,443	0.18
Dumped Pairs	4,954,514	1.6

Table S5. Genome assessment by BUSCO.

Genome	Assembly in this study (Hi-C)	Chinese alligator (*13*)	American alligator (GCF_000281125.3)
Complete BUSCOs (C)	94.3% (3724)	92.8% (3664)	94.3% (3725)
Complete and single-copy BUSCOs (S)	93.6% (3698)	92.1% (3637)	93.7% (3700)
Complete and duplicated BUSCOs (D)	0.7% (26)	0.7% (27)	0.6% (25)
Fragmented BUSCOs (F)	3.5% (139)	4.9% (192)	3.4% (134)
Missing BUSCOs (M)	2.2% (87)	2.3% (94)	2.3% (91)
Total BUSCO groups searched	3950	3950	3950

Table S6. Statistical summary for DNA-Seq alignments.

Sample	Total reads	Assembly in current study		Previous assembly (*13*)	
		Aligned concordantly exactly 1 time	Overall alignment rate	Aligned concordantly exactly 1 time	Overall alignment rate
G847-R51	15,589,399	85.55%	99.07%	85.31%	98.83%
G847-R52	13,573,694	82.04%	98.50%	81.83%	98.12%
G847-R53	11,356,605	84.27%	98.86%	84.00%	98.56%
G847-R54	13,749,331	76.23%	98.08%	76.01%	97.52%
G847-R55	15,043,060	81.30%	98.75%	81.11%	98.32%
G847-R56	10,092,036	82.29%	98.57%	82.01%	98.17%
G847-R57	12,913,739	84.66%	98.94%	84.43%	98.59%
G847-R58	16,226,029	83.88%	98.45%	83.69%	98.13%
G847-R59	14,486,362	85.81%	98.91%	85.62%	98.68%
G847-R60	13,234,546	85.87%	98.92%	85.70%	98.71%
G847-R61	14,972,343	81.43%	97.12%	81.24%	96.80%
G847-R63	3,108,719	82.57%	98.35%	82.35%	97.81%
G847-R65	11,212,522	85.73%	98.90%	85.51%	98.64%
G847-R66	10,922,498	85.16%	98.91%	84.89%	98.59%
G847-R67	15,143,684	86.01%	98.88%	85.84%	98.68%
G847-R68	12,425,285	83.43%	96.92%	83.24%	96.62%
G847-R69	12,020,258	84.43%	98.14%	84.22%	97.86%
G847-R70	15,158,407	84.29%	97.02%	84.04%	96.76%
G847-R71	15,103,939	85.87%	98.77%	85.64%	98.52%
G847-R72	13,430,343	86.35%	98.97%	86.09%	98.73%
G847-R73	11,066,377	85.81%	98.84%	85.53%	98.59%
G847-R75	11,030,560	85.28%	98.63%	85.08%	98.40%
G847-R76	15,990,019	75.59%	90.87%	75.44%	90.51%
G847-R78	12,957,782	86.03%	98.91%	85.82%	98.68%
G847-R79	14,213,176	85.31%	98.83%	85.06%	98.53%
G847-R80	14,482,724	84.68%	98.73%	84.47%	98.41%
G847-R81	14,655,528	85.69%	98.20%	85.43%	97.98%
G847-R83	11,307,959	85.67%	98.23%	85.42%	97.98%
G847-R84	12,172,736	86.11%	98.96%	85.81%	98.68%
G847-R85	15,801,865	86.35%	98.91%	86.10%	98.69%
G847-R86	4,598,969	85.47%	98.80%	85.20%	98.45%
G847-R88	14,213,475	84.31%	98.38%	84.05%	98.02%
G847-R89	14,249,620	84.22%	98.26%	83.91%	97.90%
G847-R90	10,170,255	86.01%	98.84%	85.76%	98.58%
G847-R91	12,654,914	84.03%	97.10%	83.83%	96.88%
G847-R92	14,281,647	86.48%	98.84%	86.23%	98.61%
G847-R93	12,940,711	87.81%	99.15%	87.39%	98.90%
G847-R94	13,584,575	86.86%	98.97%	86.50%	98.71%

G847-R95	12,232,997	86.76%	98.96%	86.42%	98.64%
G847-R96	13,995,810	86.53%	99.03%	86.24%	98.77%
G847-R97	12,373,297	86.48%	98.99%	86.22%	98.70%
G847-R98	11,488,236	86.13%	98.94%	85.82%	98.63%
G847-R99	15,092,796	86.32%	99.02%	86.00%	98.71%

Table S7. Statistical summary of RNA-Seq alignments.

Sample	Total reads	Assembly in current study		Previous assembly (*13*)	
		Aligned concordantly exactly 1 time	Overall alignment rate	Aligned concordantly exactly 1 time	Overall alignment rate
FD31_1	4,994,501	83.28%	91.50%	81.09%	92.14%
FD36_1	5,714,779	83.46%	91.30%	82.16%	92.76%
FD36_2	3,076,451	83.81%	90.93%	84.05%	93.79%
FD41_2	16,171,934	78.26%	86.51%	77.43%	88.72%
FD45_1	4,001,922	82.25%	90.25%	82.76%	93.43%
FD45_2	17,989,029	77.95%	86.55%	77.16%	89.01%
FD49_1	4,429,649	83.35%	90.64%	83.23%	93.37%
FD49_2	18,677,285	72.45%	80.96%	73.07%	86.05%
FD55_1	11,576,161	81.37%	89.77%	79.16%	90.43%
FH_1	3,932,568	78.30%	84.71%	82.82%	92.98%
FH_2	4,083,835	80.57%	87.25%	83.12%	93.29%
FJ1_15_6	17,915,268	79.81%	88.56%	75.50%	88.37%
FJ_16_1	15,707,586	83.41%	92.34%	76.01%	87.47%
FS1_15_6	18,652,157	72.62%	80.70%	75.76%	89.73%
FS_16_1	20,984,584	80.77%	88.99%	78.23%	89.08%
FX1_15_6	18,958,581	68.89%	76.65%	73.29%	87.30%
FX_16_1	20,449,932	81.50%	90.55%	79.60%	91.40%
MD31_1	5,949,877	84.72%	92.97%	81.72%	92.47%
MD31_2	4,375,311	84.22%	92.32%	80.91%	91.79%
MD36_1	4,444,135	82.81%	90.65%	81.51%	92.33%
MD36_2	4,702,389	83.45%	91.42%	81.38%	92.27%
MD41_1	5,790,465	83.46%	91.74%	81.29%	92.32%
MD41_2	22,495,031	75.70%	85.14%	74.61%	87.42%
MD45_1	3,815,951	80.95%	88.71%	80.72%	91.86%
MD45_2	4,788,865	82.28%	90.02%	81.70%	92.44%
MD49_1	4,885,403	79.88%	87.44%	80.84%	91.65%
MD49_2	6,374,061	81.87%	89.50%	80.90%	91.79%
MH_1	5,370,325	80.00%	87.49%	81.21%	92.36%
MH_2	6,598,986	77.08%	85.02%	78.58%	90.66%
MJ1_16_1	19,358,040	83.52%	93.45%	74.11%	88.10%
MS1_16_1	21,813,150	77.58%	86.03%	78.09%	90.51%
MX1_16_1	18,062,651	82.20%	90.53%	79.61%	90.87%

Table S8. SNPs and InDels identified by pairwise genome comparison between current assembly and other two published assemblies.

Assembly in current study	Length (bp)	Chinese alligator (*13*)				American alligator (GCF_000281125.3)			
		No. of SNPs	% of SNPs	No. of InDels	% of InDels	No. of SNPs	% of SNPs	No. of InDels	% of SNPs
LG1	252,542,828	187,605	0.0743%	61,516	0.0244%	4,630,480	1.8335%	528,362	0.2092%
LG2	239,932,975	178,173	0.0743%	55,967	0.0233%	4,389,247	1.8294%	486,680	0.2028%
LG3	231,252,442	166,640	0.0721%	50,561	0.0219%	4,261,114	1.8426%	479,861	0.2075%
LG4	219,650,141	177,857	0.0810%	55,908	0.0255%	4,084,981	1.8598%	476,804	0.2171%
LG5	160,514,988	116,924	0.0728%	38,910	0.0242%	2,940,881	1.8322%	334,056	0.2081%
LG6	145,960,254	123,579	0.0847%	38,708	0.0265%	2,706,247	1.8541%	305,937	0.2096%
LG7	152,287,032	128,516	0.0844%	38,844	0.0255%	2,822,074	1.8531%	322,440	0.2117%
LG8	149,026,059	123,308	0.0827%	38,840	0.0261%	2,707,139	1.8166%	309,956	0.2080%
LG9	154,259,617	151,061	0.0979%	42,631	0.0276%	3,413,833	2.2130%	397,863	0.2579%
LG10	101,328,341	85,025	0.0839%	25,018	0.0247%	1,995,909	1.9697%	228,293	0.2253%
LG11	84,045,948	94,613	0.1126%	24,111	0.0287%	1,655,992	1.9703%	189,018	0.2249%
LG12	83,410,568	74,443	0.0892%	22,239	0.0267%	1,678,715	2.0126%	192,795	0.2311%
LG13	77,075,714	64,379	0.0835%	19,170	0.0249%	1,525,243	1.9789%	176,967	0.2296%
LG14	72,287,167	57,059	0.0789%	17,076	0.0236%	1,447,556	2.0025%	163,996	0.2269%
LG15	67,702,914	52,181	0.0771%	16,369	0.0242%	1,370,190	2.0238%	153,822	0.2272%
LG16	61,814,854	85,148	0.1377%	19,898	0.0322%	1,193,343	1.9305%	135,657	0.2195%
Unanchored	10,542,153	40,181	0.3811%	6,627	0.0629%	148,358	1.4073%	15,997	0.1517%
Total	2,263,633,995	1,906,692	0.0842%	572,393	0.0253%	42,971,302	1.8983%	4,898,504	0.2164%

Table S9. Statistical summary of gene prediction results based on different methods.

Method	Software	Species	Gene number
Ab initio	Genscan	-	37,584
	Augustus	-	32,278
	GlimmerHMM	-	148,098
	GeneID	-	27,960
	SNAP	-	51,682
Homology-based	GeMoMa	*Danio rerio*	16,972
		Alligator mississippiensis	20,454
		Alligator sinensis	20,036
		Crocodylus porosus	15,775
		Gavialis gangeticus	15,604
RNAseq	TransDecoder	-	207,659
	GeneMarkS-T	-	43,380
	PASA	-	107,820
Integration	EVM	-	21,237

Note: “Method” indicated the strategy used for gene prediction; “Software” indicated the software used for gene prediction; Species indicated the referenced species for gene prediction; Gene number indicated the predicted gene number.

Table S10. Statistical summary for non-coding RNA genes.

	snRNA	snoRNA	rRNA	miRNA
Count	242	226	176	226
Total length (bp)	36,138	28,816	191,742	18,391
Minimum length (bp)	52	64	80	58
Maximum length (bp)	233	320	3,891	130
Average length (bp)	149	128	1,089	81

Table S11. Statistical summary of gene annotation.

Database	Annotated Number	100<=Protein length<300	Protein length>=300
GO	6,682	2,257	4,253
KEGG	13,571	3,558	9,794
KOG	13,399	3,432	9,776
TrEMBL	20,266	5,959	13,929
nr	20,598	6,176	14,024
All	20,614	6,187	14,027

Table S12. Sample information of 244 individuals for population genomics study.

Sample ID	Clean reads	Depth (x)	Type	Sample type	Sample source	Sampling year	Sampling tissue
B11_3	281,603,309	18.66	High	Captive	ACANNR	2015	Umbilical tissue
B15_13	297,670,232	19.73	High	Captive	ACANNR	2015	Umbilical tissue
B15_9	245,256,420	16.25	High	Captive	ACANNR	2015	Umbilical tissue
B16_9	270,733,028	17.94	High	Captive	ACANNR	2015	Umbilical tissue
B26_24	263,932,219	17.49	High	Captive	ACANNR	2015	Umbilical tissue
B28_16	268,704,972	17.81	High	Captive	ACANNR	2015	Umbilical tissue
B28_8	249,687,576	16.55	High	Captive	ACANNR	2015	Umbilical tissue
B2_1_2	267,719,208	17.74	High	Captive	ACANNR	2015	Umbilical tissue
B2_1_3	290,829,552	19.27	High	Captive	ACANNR	2015	Umbilical tissue
B34_10	251,600,898	16.67	High	Captive	ACANNR	2015	Umbilical tissue
B34_16	277,575,035	18.39	High	Captive	ACANNR	2015	Umbilical tissue
B3_10_2	281,620,805	18.66	High	Captive	ACANNR	2015	Umbilical tissue
B3_14_13	272,812,050	18.08	High	Captive	ACANNR	2015	Umbilical tissue
B3_14_26	267,976,882	17.76	High	Captive	ACANNR	2015	Umbilical tissue
B3_14_4	259,946,942	17.23	High	Captive	ACANNR	2015	Umbilical tissue
B3_16_3	271,519,394	17.99	High	Captive	ACANNR	2015	Umbilical tissue
B3_6_13	229,154,412	15.18	High	Captive	ACANNR	2015	Umbilical tissue
B3_6_3	276,550,824	18.33	High	Captive	ACANNR	2015	Umbilical tissue
B4_14_12	324,778,648	21.52	High	Captive	ACANNR	2015	Umbilical tissue
B4_14_4	290,889,405	19.28	High	Captive	ACANNR	2015	Umbilical tissue
B4_14_6	315,237,307	20.89	High	Captive	ACANNR	2015	Umbilical tissue
B4_3_2	265,917,986	17.62	High	Captive	ACANNR	2015	Umbilical tissue
B4_3_8	268,221,572	17.77	High	Captive	ACANNR	2015	Umbilical tissue
B4_3_9	338,118,191	22.41	High	Captive	ACANNR	2015	Umbilical tissue
B55-10	279,851,733	18.54	High	Captive	ACANNR	2015	Umbilical tissue
B55_26	245,331,964	16.26	High	Captive	ACANNR	2015	Umbilical tissue
B81_4	286,999,055	19.02	High	Captive	ACANNR	2015	Umbilical tissue
B81_6	263,057,398	17.43	High	Captive	ACANNR	2015	Umbilical tissue
B96_changle	255,473,384	16.93	High	Wild	Changle, Anhui Province	2015	Umbilical tissue
R164	24,008,431	1.59	Low	Captive	ACANNR	2015	Umbilical tissue
R86_MP	9,197,938	0.61	Low	Captive	ACANNR	2015	Umbilical tissue
R153	40,060,978	2.65	Low	Captive	ACANNR	2015	Umbilical tissue
R81	62,134,807	4.12	Low	Captive	ACANNR	2015	Umbilical tissue
R56_MP	20,184,072	1.34	Low	Captive	ACANNR	2015	Umbilical tissue
R174	46,057,445	3.05	Low	Captive	ACANNR	2015	Umbilical tissue
R98_F1	22,976,472	1.52	Low	F1	ACANNR	1999	blood
R77	59,355,364	3.93	Low	Captive	ACANNR	2015	Umbilical tissue
R84_MP	24,345,472	1.61	Low	Captive	ACANNR	2015	Umbilical tissue

R95_ZJ	24,465,994	1.62	Low	ZJ	Zhejiang Province	1999	blood
R97_F1	24,746,594	1.64	Low	F1	ACANNR	1999	blood
R91_MP	25,309,828	1.68	Low	Captive	ACANNR	2015	Umbilical tissue
R93_ZJ	25,881,422	1.72	Low	ZJ	Zhejiang Province	1999	blood
R62	62,823,604	4.16	Low	Captive	ACANNR	2015	Umbilical tissue
R94_ZJ	27,169,150	1.80	Low	ZJ	Zhejiang Province	1999	blood
R54_MP	27,498,662	1.82	Low	Captive	ACANNR	2015	Umbilical tissue
R184	51,759,098	3.43	Low	Captive	ACANNR	2015	Umbilical tissue
R96_F1	27,991,620	1.85	Low	F1	ACANNR	1999	blood
R89_MP	28,499,240	1.89	Low	Captive	ACANNR	2015	Umbilical tissue
R92_MP	28,563,294	1.89	Low	Captive	ACANNR	2015	Umbilical tissue
R80_MP	28,965,448	1.92	Low	Captive	ACANNR	2015	Umbilical tissue
R76_MP	31,980,038	2.12	Low	Captive	ACANNR	2015	Umbilical tissue
R61_SP	29,944,686	1.98	Low	Captive	ACANNR	2015	Umbilical tissue
R09	61,317,866	4.06	Low	Captive	ACANNR	2015	Umbilical tissue
R79	57,596,140	3.82	Low	Captive	ACANNR	2015	Umbilical tissue
R55_MP	30,086,120	1.99	Low	Captive	ACANNR	2015	Umbilical tissue
R71_MP	30,207,878	2.00	Low	Captive	ACANNR	2015	Umbilical tissue
R99_wild	30,185,592	2.00	Low	Wild	Exact location unknown	1999	blood
R74	56,499,078	3.74	Low	Captive	ACANNR	2015	Umbilical tissue
R155	48,388,481	3.21	Low	Captive	ACANNR	2015	Umbilical tissue
R58_CL_wild	32,452,058	2.15	Low	WIld	Changle, Anhui Province	2015	Umbilical tissue
R43	56,732,968	3.76	Low	Captive	ACANNR	2021	scale
R127	55,844,140	3.70	Low	Captive	ACANNR	2021	scale
R131	55,433,574	3.67	Low	Captive	ACANNR	2021	scale
R117	60,536,521	4.01	Low	Captive	ACANNR	2021	scale
R58	57,879,154	3.84	Low	Captive	ACANNR	2021	scale
R56	61,002,868	4.04	Low	Captive	ACANNR	2021	scale
R72	61,248,681	4.06	Low	Captive	ACANNR	2021	scale
R195	52,361,059	3.47	Low	Captive	ACANNR	2021	scale
R91	57,932,775	3.84	Low	Captive	ACANNR	2021	scale
R158	42,447,766	2.81	Low	Captive	ACANNR	2021	scale
R22	59,179,915	3.92	Low	Captive	ACANNR	2021	scale
R16	70,286,324	4.66	Low	Captive	ACANNR	2021	scale
R55	68,883,876	4.56	Low	Captive	ACANNR	2021	scale
R122	59,231,549	3.92	Low	Captive	ACANNR	2021	scale
R83	56,981,903	3.78	Low	Captive	ACANNR	2021	scale

R15	63,517,517	4.21	Low	Captive	ACANNR	2021	scale
R73	55,452,876	3.67	Low	Captive	ACANNR	2021	scale
R135	56,053,444	3.71	Low	Captive	ACANNR	2021	scale
R142	56,168,095	3.72	Low	Captive	ACANNR	2021	scale
R42	51,334,310	3.40	Low	Captive	ACANNR	2021	scale
R78	53,076,469	3.52	Low	Captive	ACANNR	2021	scale
R69	61,978,704	4.11	Low	Captive	ACANNR	2021	scale
R02	60,494,329	4.01	Low	Captive	ACANNR	2021	scale
R34	56,322,057	3.73	Low	Captive	ACANNR	2021	scale
R11	64,566,868	4.28	Low	Captive	ACANNR	2021	scale
R128	75,330,161	4.99	Low	Captive	ACANNR	2021	scale
R108	54,951,618	3.64	Low	Captive	ACANNR	2021	scale
R71	54,598,746	3.62	Low	Captive	ACANNR	2021	scale
R89	52,803,733	3.50	Low	Captive	ACANNR	2021	scale
R120	59,590,929	3.95	Low	Captive	ACANNR	2021	scale
R24	63,731,700	4.22	Low	Captive	ACANNR	2021	scale
R102	55,934,463	3.71	Low	Captive	ACANNR	2021	scale
R125	56,301,274	3.73	Low	Captive	ACANNR	2021	scale
R176	53,452,008	3.54	Low	Captive	ACANNR	2021	scale
R130	54,498,699	3.61	Low	Captive	ACANNR	2021	scale
R139	54,086,409	3.58	Low	Captive	ACANNR	2021	scale
R48	56,304,450	3.73	Low	Captive	ACANNR	2021	scale
R65	54,552,539	3.61	Low	Captive	ACANNR	2021	scale
R88	54,399,557	3.60	Low	Captive	ACANNR	2021	scale
R82	60,206,742	3.99	Low	Captive	ACANNR	2021	scale
R23	55,490,327	3.68	Low	Captive	ACANNR	2021	scale
R119	54,643,806	3.62	Low	Captive	ACANNR	2021	scale
R129	51,724,131	3.43	Low	Captive	ACANNR	2021	scale
R60	73,894,419	4.90	Low	Captive	ACANNR	2021	scale
R194	52,499,012	3.48	Low	Captive	ACANNR	2021	scale
R147	52,859,572	3.50	Low	Captive	ACANNR	2021	scale
R06	60,101,857	3.98	Low	Captive	ACANNR	2021	scale
R116	56,150,839	3.72	Low	Captive	ACANNR	2021	scale
R08	60,963,542	4.04	Low	Captive	ACANNR	2021	scale
R85	54,674,125	3.62	Low	Captive	ACANNR	2021	scale
R13	64,693,853	4.29	Low	Captive	ACANNR	2021	scale
R94	53,500,501	3.55	Low	Captive	ACANNR	2021	scale
R112	60,651,035	4.02	Low	Captive	ACANNR	2021	scale
R86	54,297,062	3.60	Low	Captive	ACANNR	2021	scale
R107	53,739,039	3.56	Low	Captive	ACANNR	2021	scale
R37	59,914,404	3.97	Low	Captive	ACANNR	2021	scale
R140	51,791,116	3.43	Low	Captive	ACANNR	2021	scale
R188	53,487,346	3.54	Low	Captive	ACANNR	2021	scale

R68	79,608,049	5.28	Low	Captive	ACANNR	2021	scale
R93	50,338,635	3.34	Low	Captive	ACANNR	2021	scale
R154	52,758,258	3.50	Low	Captive	ACANNR	2021	scale
R101	51,968,544	3.44	Low	Captive	ACANNR	2021	scale
R160	52,929,267	3.51	Low	Captive	ACANNR	2021	scale
R40	55,584,767	3.68	Low	Captive	ACANNR	2021	scale
R45	55,740,097	3.69	Low	Captive	ACANNR	2021	scale
R169	50,942,737	3.38	Low	Captive	ACANNR	2021	scale
R50	67,284,958	4.46	Low	Captive	ACANNR	2021	scale
R146	58,025,602	3.85	Low	Captive	ACANNR	2021	scale
R141	50,989,436	3.38	Low	Captive	ACANNR	2021	scale
R148	58,669,490	3.89	Low	Captive	ACANNR	2021	scale
R84	53,985,740	3.58	Low	Captive	ACANNR	2021	scale
R07	60,832,982	4.03	Low	Captive	ACANNR	2021	scale
R96	61,773,855	4.09	Low	Captive	ACANNR	2021	scale
R182	55,530,185	3.68	Low	Captive	ACANNR	2021	scale
R133	54,707,684	3.63	Low	Captive	ACANNR	2021	scale
R75	52,406,801	3.47	Low	Captive	ACANNR	2021	scale
R04	59,848,994	3.97	Low	Captive	ACANNR	2021	scale
R01	61,455,744	4.07	Low	Captive	ACANNR	2021	scale
R25	61,176,326	4.05	Low	Captive	ACANNR	2021	scale
R99	52,334,465	3.47	Low	Captive	ACANNR	2021	scale
R138	54,309,240	3.60	Low	Captive	ACANNR	2021	scale
R52	56,027,353	3.71	Low	Captive	ACANNR	2021	scale
R100	50,649,804	3.36	Low	Captive	ACANNR	2021	scale
R03	55,089,034	3.65	Low	Captive	ACANNR	2021	scale
R152	54,041,851	3.58	Low	Captive	ACANNR	2021	scale
R192	51,138,024	3.39	Low	Captive	ACANNR	2021	scale
R132	52,500,153	3.48	Low	Captive	ACANNR	2021	scale
R104	52,386,371	3.47	Low	Captive	ACANNR	2021	scale
R118	55,870,988	3.70	Low	Captive	ACANNR	2021	scale
R161	52,026,330	3.45	Low	Captive	ACANNR	2021	scale
R98	52,591,997	3.49	Low	Captive	ACANNR	2021	scale
R63	55,303,444	3.66	Low	Captive	ACANNR	2021	scale
R76	55,984,095	3.71	Low	Captive	ACANNR	2021	scale
R144	56,582,769	3.75	Low	Captive	ACANNR	2021	scale
R38	54,944,617	3.64	Low	Captive	ACANNR	2021	scale
R178	52,056,898	3.45	Low	Captive	ACANNR	2021	scale
R159	53,128,206	3.52	Low	Captive	ACANNR	2021	scale
R177	55,851,244	3.70	Low	Captive	ACANNR	2021	scale
R18	54,619,763	3.62	Low	Captive	ACANNR	2021	scale
R134	54,851,292	3.63	Low	Captive	ACANNR	2021	scale
R190	51,592,103	3.42	Low	Captive	ACANNR	2021	scale

R124	54,169,483	3.59	Low	Captive	ACANNR	2021	scale
R172	53,737,937	3.56	Low	Captive	ACANNR	2021	scale
R191	54,138,889	3.59	Low	Captive	ACANNR	2021	scale
R185	51,913,516	3.44	Low	Captive	ACANNR	2021	scale
R163	53,807,337	3.57	Low	Captive	ACANNR	2021	scale
R12	62,121,109	4.12	Low	Captive	ACANNR	2021	scale
R145	54,086,941	3.58	Low	Captive	ACANNR	2021	scale
R05	59,919,459	3.97	Low	Captive	ACANNR	2021	scale
R136	54,367,556	3.60	Low	Captive	ACANNR	2021	scale
R137	54,896,549	3.64	Low	Captive	ACANNR	2021	scale
R92	57,530,545	3.81	Low	Captive	ACANNR	2021	scale
R39	60,632,528	4.02	Low	Captive	ACANNR	2021	scale
R111	55,466,004	3.68	Low	Captive	ACANNR	2021	scale
R61	53,462,671	3.54	Low	Captive	ACANNR	2021	scale
R35	54,919,273	3.64	Low	Captive	ACANNR	2021	scale
R46	54,953,817	3.64	Low	Captive	ACANNR	2021	scale
R80	58,953,601	3.91	Low	Captive	ACANNR	2021	scale
R180	54,031,964	3.58	Low	Captive	ACANNR	2021	scale
R187	55,281,359	3.66	Low	Captive	ACANNR	2021	scale
R123	57,283,264	3.80	Low	Captive	ACANNR	2021	scale
R54	54,634,215	3.62	Low	Captive	ACANNR	2021	scale
R49	62,629,544	4.15	Low	Captive	ACANNR	2021	scale
R105	54,803,149	3.63	Low	Captive	ACANNR	2021	scale
R121	63,597,797	4.21	Low	Captive	ACANNR	2021	scale
R33	61,635,305	4.08	Low	Captive	ACANNR	2021	scale
R67	59,225,444	3.92	Low	Captive	ACANNR	2021	scale
R167	57,466,384	3.81	Low	Captive	ACANNR	2021	scale
R53	57,936,805	3.84	Low	Captive	ACANNR	2021	scale
R157	59,606,738	3.95	Low	Captive	ACANNR	2021	scale
R193	61,127,312	4.05	Low	Captive	ACANNR	2021	scale
R114	54,890,982	3.64	Low	Captive	ACANNR	2021	scale
R181	55,525,163	3.68	Low	Captive	ACANNR	2021	scale
R36	56,926,542	3.77	Low	Captive	ACANNR	2021	scale
R95	62,776,740	4.16	Low	Captive	ACANNR	2021	scale
R189	55,564,295	3.68	Low	Captive	ACANNR	2021	scale
R21	65,082,109	4.31	Low	Captive	ACANNR	2021	scale
R113	56,068,082	3.72	Low	Captive	ACANNR	2021	scale
R115	60,444,511	4.01	Low	Captive	ACANNR	2021	scale
R110	63,762,548	4.23	Low	Captive	ACANNR	2021	scale
R44	57,641,864	3.82	Low	Captive	ACANNR	2021	scale
R17	59,293,584	3.93	Low	Captive	ACANNR	2021	scale
R156	58,056,759	3.85	Low	Captive	ACANNR	2021	scale
R126	59,822,958	3.96	Low	Captive	ACANNR	2021	scale

R165	56,840,161	3.77	Low	Captive	ACANNR	2021	scale
R31	61,225,400	4.06	Low	Captive	ACANNR	2021	scale
R168	57,961,541	3.84	Low	Captive	ACANNR	2021	scale
R183	57,397,703	3.80	Low	Captive	ACANNR	2021	scale
R51	67,630,845	4.48	Low	Captive	ACANNR	2021	scale
R59	59,489,925	3.94	Low	Captive	ACANNR	2021	scale
R149	61,965,289	4.11	Low	Captive	ACANNR	2021	scale
R97	58,412,801	3.87	Low	Captive	ACANNR	2021	scale
R28	60,578,617	4.01	Low	Captive	ACANNR	2021	scale
R151	58,577,018	3.88	Low	Captive	ACANNR	2021	scale
R32	64,297,118	4.26	Low	Captive	ACANNR	2021	scale
R14	60,407,982	4.00	Low	Captive	ACANNR	2021	scale
R29	58,511,898	3.88	Low	Captive	ACANNR	2021	scale
R175	58,010,133	3.84	Low	Captive	ACANNR	2021	scale
R64	59,031,939	3.91	Low	Captive	ACANNR	2021	scale
R26	62,902,678	4.17	Low	Captive	ACANNR	2021	scale
R103	62,454,193	4.14	Low	Captive	ACANNR	2021	scale
R41	61,802,156	4.10	Low	Captive	ACANNR	2021	scale
R171	63,599,962	4.21	Low	Captive	ACANNR	2021	scale
R30	65,022,431	4.31	Low	Captive	ACANNR	2021	scale
R87	60,789,053	4.03	Low	Captive	ACANNR	2021	scale
R186	59,335,417	3.93	Low	Captive	ACANNR	2021	scale
R143	62,304,951	4.13	Low	Captive	ACANNR	2021	scale
R109	64,140,789	4.25	Low	Captive	ACANNR	2021	scale
R90	63,470,603	4.21	Low	Captive	ACANNR	2021	scale
R170	63,502,819	4.21	Low	Captive	ACANNR	2021	scale
R162	61,717,375	4.09	Low	Captive	ACANNR	2021	scale
R166	61,369,178	4.07	Low	Captive	ACANNR	2021	scale
R19	65,409,065	4.33	Low	Captive	ACANNR	2021	scale
R173	62,172,633	4.12	Low	Captive	ACANNR	2021	scale
R150	63,272,190	4.19	Low	Captive	ACANNR	2021	scale
R10	66,664,929	4.42	Low	Captive	ACANNR	2021	scale
R27	73,374,898	4.86	Low	Captive	ACANNR	2021	scale
R57	65,373,645	4.33	Low	Captive	ACANNR	2021	scale
R106	67,093,824	4.45	Low	Captive	ACANNR	2021	scale
R179	66,611,727	4.41	Low	Captive	ACANNR	2021	scale
R66	73,193,018	4.85	Low	Captive	ACANNR	2021	scale
R20	71,293,222	4.72	Low	Captive	ACANNR	2021	scale
R47	70,270,053	4.66	Low	Captive	ACANNR	2021	scale
R70	79,959,472	5.30	Low	Captive	ACANNR	2021	scale

ACANNR: Anhui Chinese Alligator National Nature Reserve.

Table S13. Genome-wide heterozygosity of 29 individuals sequenced at normal depth.

Sample ID	Heterozygous SNP sites	Heterozygosity
B11_3	361,401	1.5966E-04
B15_13	426,305	1.8833E-04
B15_9	429,637	1.8980E-04
B16_9	461,258	2.0377E-04
B26_24	448,584	1.9817E-04
B28_16	473,664	2.0925E-04
B28_8	382,090	1.6879E-04
B2_1_2	462,429	2.0429E-04
B2_1_3	424,015	1.8732E-04
B34_10	349,045	1.5420E-04
B34_16	421,679	1.8628E-04
B3_10_2	404,762	1.7881E-04
B3_14_13	434,769	1.9207E-04
B3_14_26	427,328	1.8878E-04
B3_14_4	439,395	1.9411E-04
B3_16_3	415,690	1.8364E-04
B3_6_13	394,943	1.7447E-04
B3_6_3	423,151	1.8693E-04
B4_14_12	492,267	2.1747E-04
B4_14_4	507,739	2.2430E-04
B4_14_6	500,618	2.2116E-04
B4_3_2	355,991	1.5727E-04
B4_3_8	435,042	1.9219E-04
B4_3_9	410,406	1.8130E-04
B55.10	432,430	1.9103E-04
B55_26	471,464	2.0828E-04
B81_4	431,859	1.9078E-04
B81_6	393,479	1.7383E-04
B96_changle	272,627	1.2044E-04

Table S14. Statistical summary for ROH across 29 individuals sequenced at normal depth.

Sample ID	NSEG	KB	KBAVG	Type	FROH (%)
B11_3	358	928,514	2,593.62	Captivity	41.02
B15_13	366	894,997	2,445.35	Captivity	39.54
B15_9	316	682,200	2,158.86	Captivity	30.14
B16_9	291	606,003	2,082.48	Captivity	26.77
B26_24	306	680,222	2,222.95	Captivity	30.05
B28_16	264	548,322	2,076.98	Captivity	24.22
B28_8	402	903,463	2,247.42	Captivity	39.91
B2_1_2	277	581,877	2,100.64	Captivity	25.71
B2_1_3	341	829,756	2,433.30	Captivity	36.66
B34_10	417	974,052	2,335.86	Captivity	43.03
B34_16	285	632,739	2,220.14	Captivity	27.95
B3_10_2	341	933,510	2,737.57	Captivity	41.24
B3_14_13	370	757,901	2,048.38	Captivity	33.48
B3_14_26	345	830,879	2,408.35	Captivity	36.71
B3_14_4	349	806,461	2,310.78	Captivity	35.63
B3_16_3	331	770,450	2,327.64	Captivity	34.04
B3_6_13	379	883,558	2,331.29	Captivity	39.03
B3_6_3	410	860,138	2,097.90	Captivity	38.00
B4_14_12	281	657,417	2,339.56	Captivity	29.04
B4_14_4	203	444,288	2,188.61	Captivity	19.63
B4_14_6	230	450,149	1,957.17	Captivity	19.89
B4_3_2	372	792,421	2,130.16	Captivity	35.01
B4_3_8	288	485,507	1,685.79	Captivity	21.45
B4_3_9	390	795,261	2,039.13	Captivity	35.13
B55-10	334	790,092	2,365.54	Captivity	34.90
B55_26	326	722,162	2,215.22	Captivity	31.90
B81_4	288	642,647	2,231.41	Captivity	28.39
B81_6	357	841,401	2,356.87	Captivity	37.17
B96_changle	488	1,277,700	2,618.25	Wild	56.44

NSEG: number of segments; KBAVG: average ROH in kb.

Table S15. FHOM inbreeding coefficients of 29 individuals sequenced at normal depth.

Sample ID	O(HOM)	E(HOM)	N(NM)	FHOM	Type	Source
B11_3	888,075	861,100	1,248,983	0.0694	Captive	ACANNR
B15_13	827,384	864,300	1,253,134	-0.0950	Captive	ACANNR
B15_9	821,198	862,300	1,250,311	-0.1058	Captive	ACANNR
B16_9	790,253	862,700	1,251,003	-0.1867	Captive	ACANNR
B26_24	797,948	859,000	1,245,979	-0.1578	Captive	ACANNR
B28_16	777,757	862,500	1,250,839	-0.2183	Captive	ACANNR
B28_8	867,615	861,300	1,249,194	0.0162	Captive	ACANNR
B2_1_2	788,062	861,900	1,249,902	-0.1903	Captive	ACANNR
B2_1_3	827,326	862,500	1,250,744	-0.0906	Captive	ACANNR
B34_10	900,227	861,100	1,248,841	0.1008	Captive	ACANNR
B34_16	829,537	862,500	1,250,716	-0.0848	Captive	ACANNR
B3_10_2	845,082	861,400	1,249,283	-0.0422	Captive	ACANNR
B3_14_13	815,530	861,800	1,249,757	-0.1192	Captive	ACANNR
B3_14_26	824,215	862,700	1,251,003	-0.0991	Captive	ACANNR
B3_14_4	810,714	861,700	1,249,596	-0.1314	Captive	ACANNR
B3_16_3	831,822	859,700	1,246,969	-0.0720	Captive	ACANNR
B3_6_13	850,227	858,000	1,244,663	-0.0201	Captive	ACANNR
B3_6_3	827,491	862,100	1,250,108	-0.0891	Captive	ACANNR
B4_14_12	760,306	863,400	1,252,016	-0.2653	Captive	ACANNR
B4_14_4	743,494	862,400	1,250,643	-0.3064	Captive	ACANNR
B4_14_6	752,118	863,500	1,252,097	-0.2867	Captive	ACANNR
B4_3_2	892,533	860,500	1,248,048	0.0828	Captive	ACANNR
B4_3_8	816,694	862,900	1,251,235	-0.1188	Captive	ACANNR
B4_3_9	843,307	864,300	1,253,185	-0.0540	Captive	ACANNR
B55-10	816,676	861,000	1,248,537	-0.1144	Captive	ACANNR
B55_26	774,794	858,800	1,245,612	-0.2173	Captive	ACANNR
B81_4	819,585	862,500	1,250,866	-0.1106	Captive	ACANNR
B81_6	854,238	859,900	1,247,175	-0.0147	Captive	ACANNR
B96_changle	975,916	860,300	1,247,997	0.2981	Wild	Changle, Anhui Province

Table S16. Wild individual B96_changle private ROHs compared to 28 captive individuals.

Chr	Start	End
LG01	7,043	586,798
LG01	3,601,473	3,694,614
LG02	53,958,410	54,028,062
LG02	94,140,046	94,228,185
LG02	106,672,992	106,678,968
LG02	148,897,246	148,921,187
LG03	76,808,352	76,814,723
LG03	187,121,944	187,150,105
LG04	69,132,172	69,132,243
LG04	204,266,162	204,416,512
LG05	1,549,274	1,549,292
LG05	16,554,121	16,554,126
LG05	22,971,856	23,416,960
LG05	131,588,048	131,588,320
LG07	119,892,027	119,892,028
LG08	136,583,392	136,606,936
LG09	38,928,745	38,928,899
LG09	43,873,826	43,875,043
LG09	60,683,432	60,683,707
LG09	97,406,260	97,407,619
LG09	99,544,032	99,544,150
LG09	120,816,744	121,005,721
LG09	139,603,726	139,610,365
LG11	38,197,087	38,292,656
LG11	41,343,937	41,610,162
LG11	82,971,662	82,974,150
LG11	84,033,127	84,034,210
LG12	16,672,943	16,673,345
LG12	27,207,410	28,393,990
LG13	23,287,975	23,288,649
LG13	69,209,811	69,209,841

Table S17. Gene located in the wild individual B96_changle private ROHs compared to 28 captive individuals.

Chr	Start	End	Gene
LG01	234,807	266,878	EVM0004580
LG01	521,449	525,128	EVM0006272
LG01	21,657	182,410	EVM0002891
LG01	10,278	17,679	EVM0009623
LG01	204,680	225,896	EVM0000013
LG01	374,997	379,815	EVM0001880
LG01	290,936	319,045	EVM0013715
LG01	406,916	411,574	EVM0016417
LG01	498,972	514,777	EVM0001574
LG01	457,006	459,580	EVM0003725
LG01	441,165	454,658	EVM0017306
LG01	419,947	426,684	EVM0020553
LG01	554,762	573,252	EVM0013662
LG01	7,121	7,942	EVM0020658
LG01	199,706	202,044	EVM0005336
LG01	283,607	287,777	EVM0009455
LG01	278,717	280,583	EVM0011727
LG01	320,770	323,280	EVM0020077
LG01	333,520	338,356	EVM0005636
LG01	338,401	339,465	EVM0001624
LG01	353,502	357,523	EVM0007908
LG01	360,979	367,118	EVM0000864
LG01	385,680	388,043	EVM0009456
LG01	400,191	403,822	EVM0015468
LG01	405,121	406,613	EVM0005666
LG01	411,816	419,172	EVM0015528
LG01	437,900	439,633	EVM0003285
LG01	428,325	437,248	EVM0008195
LG01	460,128	464,120	EVM0004682
LG01	532,228	532,950	EVM0021048
LG01	537,134	539,364	EVM0014650
LG01	585,715	586,305	EVM0018802
LG02	148,844,645	148,899,922	EVM0006898
LG02	94,223,041	94,282,840	EVM0013405
LG02	93,991,976	94,188,556	EVM0005047
LG03	187,080,534	187,126,409	EVM0013997
LG04	204,302,072	204,447,986	EVM0020101
LG05	23,186,929	23,237,306	EVM0016203
LG05	23,149,173	23,154,054	EVM0007164
LG05	23,379,942	23,419,590	EVM0003265

LG05	23,054,014	23,063,942	EVM0014510
LG05	23,087,125	23,098,157	EVM0012824
LG05	23,112,506	23,115,663	EVM0011455
LG05	23,128,467	23,129,321	EVM0015514
LG05	23,123,183	23,126,049	EVM0017112
LG05	23,143,685	23,147,219	EVM0015605
LG05	23,154,057	23,159,363	EVM0007692
LG05	23,175,293	23,176,416	EVM0007789
LG05	23,328,554	23,329,501	EVM0003906
LG05	23,359,487	23,362,616	EVM0011844
LG05	23,366,532	23,372,099	EVM0009498
LG09	38,927,433	38,931,755	EVM0001022
LG09	120,976,851	120,980,268	EVM0007710
LG09	120,836,887	120,866,399	EVM0013643
LG09	120,784,326	120,826,496	EVM0013910
LG09	120,922,755	120,970,996	EVM0004645
LG09	99,541,690	99,545,322	EVM0018794
LG09	120,881,689	120,884,241	EVM0002924
LG09	120,984,742	120,987,045	EVM0011661
LG09	120,997,855	121,009,940	EVM0012640
LG09	139,593,802	139,606,566	EVM0007619
LG11	38,270,644	38,285,332	EVM0013807
LG11	38,178,235	38,219,612	EVM0004470
LG11	38,252,541	38,257,033	EVM0018282
LG11	38,221,271	38,222,188	EVM0004433
LG11	38,234,978	38,237,362	EVM0014648
LG11	38,266,197	38,268,794	EVM0001763
LG12	16,635,002	16,673,181	EVM0012093
LG12	27,216,273	27,247,782	EVM0014934
LG12	27,263,020	27,289,045	EVM0010487
LG12	27,289,085	27,305,646	EVM0016698
LG12	27,441,536	27,448,140	EVM0002280
LG12	27,550,307	27,577,497	EVM0009567
LG12	27,599,791	27,631,625	EVM0013345
LG12	28,235,054	28,269,752	EVM0000872
LG12	28,358,220	28,387,038	EVM0009526
LG12	28,096,576	28,098,020	EVM0013927
LG12	28,168,185	28,170,591	EVM0013785
LG12	28,203,395	28,204,054	EVM0009617
LG13	23,186,051	23,363,233	EVM0020321

Table S18. Genome-wide heterozygosity among Aves, Crocodylia, Mammalia, Squamata and Testudines.

Species	Common name	Heterozygosity	Sources
Lynx pardinus	Iberian lynx	0.0001020	(*80*)
Alligator sinensis	Chinese alligator	0.0001200	(*15*)
Lipotes vexillifer	Baiji	0.0001220	(*81*)
Alligator mississippiensis	American alligator	0.0001360	(*82*)
Urocyon littoralis	Island fox	0.0001550	(*83*)
Acinonyx jubatus	African cheetah	0.0002000	(*84*)
Gavialis gangeticus	Indian gharial	0.0002170	(*82*)
Panthera uncia	Snow leopard	0.0002300	(*85*)
Crocodylus porosus	Saltwater crocodile	0.0003600	(*82*)
Shinisaurus crocodilurus	Chinese crocodile lizard	0.0003850	(*86*)
Nipponia nippon	Crested ibis_contemporary	0.0004370	(*87*)
Gorilla beringei graueri	Eastern lowland gorilla	0.0006430	(*21*)
Gorilla beringei beringei	Eastern mountain gorilla	0.0006480	(*21*)
Nipponia nippon	Crested ibis_history	0.0007360	(*87*)
Puma concolor	Puma	0.0008480	(*88*)
Manis javanica	Malayan pangolin	0.0008530	(*89*)
Deinagkistrodon acutus	five-pacer viper	0.0010000	(*90*)
Mammuthus primigenius	Woolly mammoth	0.0011300	(*91*)
Ailuropoda melanoleuca qinlingensis	Giant panda QLI subspecies	0.0011300	(*92*)
Ailuropoda melanoleuca qinlingensis	Giant panda SC subspecies	0.0012200	(*92*)
Rafetus swinhoei	Swinhoes soft-shelled turtle	0.0012300	(*93*)
Manis pentadactyla	Chinese pangolin	0.0012700	(*89*)
Nipponia nippon	Crested ibis	0.0004300	(*94*)
Egretta garzetta	Little egret	0.0025100	(*94*)
Pelecanus crispus	Dalmatian pelican	0.0006000	(*94*)
Phalacrocorax carbo	Great black cormorant	0.0013900	(*94*)
Nestor notabilis	Kea	0.0009100	(*94*)
Melopsittacus undulatus	Budgerigar	0.0043100	(*94*)
Haliaeetus leucocephalus	Bald eagle	0.0004300	(*94*)
Haliaeetus albicilla	White-tailed Eagle	0.0004000	(*94*)
Cathartes aura	Turkey vulture	0.0011800	(*94*)
Crossoptilon mantchuricum	Brown eared pheasant (Western)	0.0000733	(*29*)
Crossoptilon mantchuricum	Brown eared pheasant (Eastern)	0.0000873	(*29*)
Crossoptilon mantchuricum	Brown eared pheasant (Center)	0.0001370	(*29*)
Falco peregrinus	Peregrine	0.0007000	(*95*)
Falco cherrug	Saker falcon	0.0008000	(*95*)
Taeniopygia guttata	Zebra finch	0.0014000	(*96*)
Ectopistes migratorius	Passenger pigeon	0.0012000	(*97*)
Taeniopygia guttata	Zebra finch	0.0014000	(*96*)

Meleagris gallopavo	Wild turkey	0.0023700	(*98*)
Apteryx mantelli	Kiwi	0.0015000	(*99*)
Ficedula albicolis	Collared flycatcher	0.0032389	(*100*)
Anas platyrhynchos	Mallard	0.0026100	(*101*)
Gallus gallus	Chicken	0.0058800	(*98*)
Meleagris gallopavo	Turkey	0.0010700	(*102*)
Crossoptilon auritum	Blue eared pheasant	0.0008250	(*29*)
Ficedula albicollis	Collared flycatcher	0.0038000	(*98*)

Supplementary Materials

Data S1. SNPs between the wild individual (B96_changle) and the captive population (n =28) with normal sequencing depth.
Data S2. ROHs identified in 29 normal-depth sequenced individuals.

REFERENCE AND NOTES

1. G. Caughley, Directions in conservation biology. *J. Anim. Ecol.* **63**, 215–244 (1994).

2. E. E. Armstrong, A. Khan, R. W. Taylor, A. Gouy, G. Greenbaum, A. Thiéry, J. T. Kang, S. A. Redondo, S. Prost, G. Barsh, C. Kaelin, S. Phalke, A. Chugani, M. Gilbert, D. Miquelle, A. Zachariah, U. Borthakur, A. Reddy, E. Louis, O. A. Ryder, Y. V. Jhala, D. Petrov, L. Excoffier, E. Hadly, U. Ramakrishnan, Recent evolutionary history of tigers highlights contrasting roles of genetic drift and selection. *Mol. Biol. Evol.* **38**, 2366–2379 (2021).

3. P. Pečnerová, G. Garcia-Erill, X. Liu, C. Nursyifa, R. K. Waples, C. G. Santander, L. Quinn, P. Frandsen, J. Meisner, F. F. Stæger, M. S. Rasmussen, A. Brüniche-Olsen, C. H. F. Jørgensen, R. R. da Fonseca, H. R. Siegismund, A. Albrechtsen, R. Heller, I. Moltke, K. Hanghøj, High genetic diversity and low differentiation reflect the ecological versatility of the African leopard. *Curr. Biol.* **31**, 1862–1871.e5(2021).

4. J. von Seth, N. Dussex, D. Díez-del-Molino, T. van der Valk, V. E. Kutschera, M. Kierczak, C. C. Steiner, S. Liu, M. Thomas P. Gilbert, M.-H. S. Sinding, S. Prost, K. Guschanski, S. K. S. S. Nathan, S. Brace, Y. L. Chan, C. W. Wheat, P. Skoglund, O. A. Ryder, B. Goossens, A. Götherström, L. Dalén, Genomic insights into the conservation status of the world's last remaining Sumatran rhinoceros populations. *Nat. Commun.* **12**, 2393 (2021).

5. T. Van Der Valk, D. Díez-del-Molino, T. Marques-Bonet, K. Guschanski, L. Dalén, Historical genomes reveal the genomic consequences of recent population decline in eastern gorillas. *Curr. Biol.* **29**, 165–170.e6 (2019).

6. H. De Kort, J. G. Prunier, S. Ducatez, O. Honnay, M. Baguette, V. M. Stevens, S. Blanchet, Life history, climate and biogeography interactively affect worldwide genetic diversity of plant and animal populations. *Nat. Commun.* **12**, 516 (2021).

7. G. Huang, X. Ping, W. Xu, Y. Hu, J. Chang, R. R. Swaisgood, J. Zhou, X. Zhan, Z. Zhang, Y. Nie, J. Cui, M. Bruford, Z. Zhang, B. Li, L. Zhang, Z. Lv, F. Wei, Wildlife conservation and management in China: Achievements, challenges and perspectives. *Natl. Sci. Rev.* **8**, nwab042 (2021).

8. M. Bosse, H. J. Megens, M. F. Derks, Á. M. de Cara, M. A. Groenen, Deleterious alleles in the context of domestication, inbreeding, and selection. *Evol. Appl.* **12**, 6–17 (2019).

9. G. Ceballos, P. R. Ehrlich, R. Dirzo, Biological annihilation via the ongoing sixth mass extinction signaled by vertebrate population losses and declines. *Proc. Natl. Acad. Sci. U.S.A.* **114**, E6089–E6096 (2017).

10. N. M. Haddad, L. A. Brudvig, J. Clobert, K. F. Davies, A. Gonzalez, R. D. Holt, T. E. Lovejoy, J. O. Sexton, M. P. Austin, C. D. Collins, W. M. Cook, E. I. Damschen, R. M. Ewers, B. L. Foster, C. N. Jenkins, A. J. King, W. F. Laurance, D. J. Levey, C. R. Margules, B. A. Melbourne, A. O. Nicholls, J. L. Orrock, D.-X. Song, J. R. Townshend, Habitat fragmentation and its lasting impact on Earth's ecosystems. *Sci. Adv.* **1**, e1500052 (2015).

11. X. Wu, C. Gu, J. Zhu, *Comprehensive study on Anhui Alligator National Nature Reserve. In Chinese* (Hefei University of Technology Press, Hefei, 2008).

12. M. M. Cohen, C. Gans, The chromosomes of the order Crocodilia. *Cytogenetics* **9**, 81–105 (1970).

13. Q.-H. Wan, S.-K. Pan, L. Hu, Y. Zhu, P.-W. Xu, J.-Q. Xia, H. Chen, G.-Y. He, J. He, X.-W. Ni, H.-L. Hou, S.-G. Liao, H.-Q. Yang, Y. Chen, S.-K. Gao, Y.-F. Ge, C.-C. Cao, P.-F. Li, L.-M. Fang, L. Liao, S. Zhang, M.-Z. Wang, W. Dong, S.-G. Fang, Genome analysis and signature discovery for diving and sensory properties of the endangered Chinese alligator. *Cell Res.* **23**, 1091–1105 (2013).

14. G. Parra, K. Bradnam, I. Korf, CEGMA: A pipeline to accurately annotate core genes in eukaryotic genomes. *Bioinformatics* **23**, 1061–1067 (2007).

15. S. Yang, T. Lan, Y. Zhang, Q. Wang, H. Li, N. Dussex, S. K. Sahu, M. Shi, M. Hu, Y. Zhu, J. Cao, L. Liu, J. Lin, Q.-H. Wan, H. Liu, S.-G. Fang, Genomic investigation of the Chinese alligator reveals wild–extinct genetic diversity and genomic consequences of their continuous decline. *Mol. Ecol. Resour.* **23**, 294–311 (2023).

16. T. S. Korneliussen, I. Moltke, NgsRelate: A software tool for estimating pairwise relatedness from next-generation sequencing data. *Bioinformatics* **31**, 4009–4011 (2015).

17. S. S. Hauser, S. J. Galla, A. S. Putnam, T. E. Steeves, E. K. Latch, Comparing genome-based estimates of relatedness for use in pedigree-based conservation management. *Mol. Ecol. Resour.* **22**, 2546–2558 (2022).

18. A. K. Nøhr, K. Hanghøj, G. Garcia-Erill, Z. Li, I. Moltke, A. Albrechtsen, NGSremix: A software tool for estimating pairwise relatedness between admixed individuals from next-generation sequencing data. *G3 (Bethesda)* **11**, jkab174 (2021).

19. M. Kardos, M. Åkesson, T. Fountain, Ø. Flagstad, O. Liberg, P. Olason, H. Sand, P. Wabakken, C. Wikenros, H. Ellegren, Genomic consequences of intensive inbreeding in an isolated wolf population. *Nat. Ecol. Evol.* **2**, 124–131 (2018).

20. T. J. Pemberton, D. Absher, M. W. Feldman, R. M. Myers, N. A. Rosenberg, J. Z. Li, Genomic patterns of homozygosity in worldwide human populations. *Am. J. Hum. Genet.* **91**, 275–292 (2012).

21. Y. Xue, J. Prado-Martinez, P. H. Sudmant, V. Narasimhan, Q. Ayub, M. Szpak, P. Frandsen, Y. Chen, B. Yngvadottir, D. N. Cooper, M. D. Manuel, J. Hernandez-Rodriguez, I. Lobon, H. R. Siegismund, L. Pagani, M. A. Quail, C. Hvilsom, A. Mudakikwa, E. E. Eichler, M. R. Cranfield, T. Marques-Bonet, C. Tyler-Smith, A. Scally, Mountain gorilla genomes reveal the impact of long-term population decline and inbreeding. *Science* **348**, 242–245 (2015).

22. S. Schiffels, K. Wang, MSMC and MSMC2: The multiple sequentially markovian coalescent. *Methods Mol. Biol.*, 147–166 (2020).

23. M. Barbato, P. Orozco-terWengel, M. Tapio, M. W. Bruford, SNeP: A tool to estimate trends in recent effective population size trajectories using genome-wide SNP data. *Front. Genet.* **6**, 109 (2015).

24. E. Santiago, I. Novo, A. F. Pardiñas, M. Saura, J. Wang, A. Caballero, Recent demographic history inferred by high-resolution analysis of linkage disequilibrium. *Mol. Biol. Evol.* **37**, 3642–3653 (2020).

25. M. E. Watanabe, The Chinese alligator: Is farming the last hope? *Oryx* **17**, 176–181 (1983).

26. J. Thorbjarnarson, X. Wang, S. Ming, L. He, Y. Ding, Y. Wu, S. T. McMurry, Wild populations of the Chinese alligator approach extinction. *Biol. Conserv.* **103**, 93–102 (2002).

27. Y. Ding, X. Wang, Factors influencing the population status of wild Chinese alligators (*Alligator sinensis*). *Biodivers. Sci.* **12**, 324–332 (2004).

28. R. Frankham, C. J. A. Bradshaw, B. W. Brook, Genetics in conservation management: Revised recommendations for the 50/500 rules, Red List criteria and population viability analyses. *Biol. Conserv.* **170**, 56–63 (2014).

29. P. Wang, J. T. Burley, Y. Liu, J. Chang, D. Chen, Q. Lu, S.-H. Li, X. Zhou, S. Edwards, Z. Zhang, Genomic consequences of long-term population decline in brown eared pheasant. *Mol. Biol. Evol.* **38**, 263–273 (2021).

30. T. Pan, H. Wang, S. Duan, I. Ali, P. Yan, R. Cai, M. Wang, J. Zhang, H. Zhang, B. Zhang, X. Wu, Historical population decline and habitat loss in a critically endangered species, the Chinese alligator (*Alligator sinensis*). *Glob. Ecol. Conserv.* **20**, e00692 (2019).

31. A. García-Dorado, On the consequences of ignoring purging on genetic recommendations for minimum viable population rules. *Heredity* **115**, 185–187 (2015).

32. P. W. Hedrick, A. Garcia-Dorado, Understanding inbreeding depression, purging, and genetic rescue. *Trends Ecol. Evol.* **31**, 940–952 (2016).

33. G. A. A. Silva, A. M. Harder, K. B. Kirksey, S. Mathur, J. R. Willoughby, Detectability of runs of homozygosity is influenced by analysis parameters as well as population-specific demographic history. *PLoS Comput. Biol.* **20**, e1012566 (2024).

34. S. P. Rao, M. H. Huntley, N. C. Durand, E. K. Stamenova, I. D. Bochkov, J. T. Robinson, A. L. Sanborn, I. Machol, A. D. Omer, E. S. Lander, E. L. Aiden, A 3D map of the human genome at kilobase resolution reveals principles of chromatin looping. *Cell* **159**, 1665–1680 (2014).

35. S. Koren, B. P. Walenz, K. Berlin, J. R. Miller, N. H. Bergman, A. M. Phillippy, Canu: Scalable and accurate long-read assembly via adaptive *k*-mer weighting and repeat separation. *Genome Res.* **27**, 722–736 (2017).

36. R. Vaser, I. Sović, N. Nagarajan, M. Šikić, Fast and accurate de novo genome assembly from long uncorrected reads. *Genome Res.* **27**, 737–746 (2017).

37. B. J. Walker, T. Abeel, T. Shea, M. Priest, A. Abouelliel, S. Sakthikumar, C. A. Cuomo, Q. Zeng, J. Wortman, S. K. Young, A. M. Earl, Pilon: An integrated tool for comprehensive microbial variant detection and genome assembly improvement. *PLOS ONE* **9**, e112963 (2014).

38. N. Servant, N. Varoquaux, B. R. Lajoie, E. Viara, C.-J. Chen, J.-P. Vert, E. Heard, J. Dekker, E. Barillot, HiC-Pro: An optimized and flexible pipeline for Hi-C data processing. *Genome Biol.* **16**, 259 (2015).

39. H. Li, R. Durbin, Fast and accurate short read alignment with Burrows-Wheeler transform. *Bioinformatics* **25**, 1754–1760 (2009).

40. J. N. Burton, A. Adey, R. P. Patwardhan, R. Qiu, J. O. Kitzman, J. Shendure, Chromosome-scale scaffolding of de novo genome assemblies based on chromatin interactions. *Nat. Biotechnol.* **31**, 1119–1125 (2013).

41. F. A. Simão, R. M. Waterhouse, P. Ioannidis, E. V. Kriventseva, E. M. Zdobnov, BUSCO: Assessing genome assembly and annotation completeness with single-copy orthologs. *Bioinformatics* **31**, 3210–3212 (2015).

42. D. Kim, B. Langmead, S. L. Salzberg, HISAT: A fast spliced aligner with low memory requirements. *Nat. Methods* **12**, 357–360 (2015).

43. G. Marçais, A. L. Delcher, A. M. Phillippy, R. Coston, S. L. Salzberg, A. Zimin, MUMmer4: A fast and versatile genome alignment system. *PLoS Comput. Biol.* **14**, e1005944 (2018).

44. Z. Xu, H. Wang, LTR_FINDER: An efficient tool for the prediction of full-length LTR retrotransposons. *Nucleic Acids Res.* **35**, W265–W268 (2007).

45. A. L. Price, N. C. Jones, P. A. Pevzner, De novo identification of repeat families in large genomes. *Bioinformatics* **21**, i351–i358 (2005).

46. C. Hoede, S. Arnoux, M. Moisset, T. Chaumier, O. Inizan, V. Jamilloux, H. Quesneville, PASTEC: An automatic transposable element classification tool. *PLOS ONE* **9**, e91929 (2014).

47. M. Tarailo-Graovac, N. Chen, Using RepeatMasker to identify repetitive elements in genomic sequences. *Curr. Protoc. Bioinformatics* **25**, 4.10.1 –14.10.14 (2009).

48. C. Burge, S. Karlin, Prediction of complete gene structures in human genomic DNA. *J. Mol. Biol.* **268**, 78–94 (1997).

49. M. Stanke, S. Waack, Gene prediction with a hidden Markov model and a new intron submodel. *Bioinformatics* **19**, ii215–ii225 (2003).

50. W. H. Majoros, M. Pertea, S. L. Salzberg, TigrScan and GlimmerHMM: Two open source ab initio eukaryotic gene-finders. *Bioinformatics* **20**, 2878–2879 (2004).

51. E. Blanco, G. Parra, R. Guigó, Using geneid to identify genes. *Curr. Protoc. Bioinformatics* **18**, 4.3.1 –4.3.28 (2007).

52. I. Korf, Gene finding in novel genomes. *BMC Bioinformatics* **5**, 59 (2004).

53. J. Keilwagen, M. Wenk, J. L. Erickson, M. H. Schattat, J. Grau, F. Hartung, Using intron position conservation for homology-based gene prediction. *Nucleic Acids Res.* **44**, e89 (2016).

54. J. Keilwagen, F. Hartung, M. Paulini, S. O. Twardziok, J. Grau, Combining RNA-seq data and homology-based gene prediction for plants, animals and fungi. *BMC Bioinformatics* **19**, 189 (2018).

55. M. Pertea, G. M. Pertea, C. M. Antonescu, T. C. Chang, J. T. Mendell, S. L. Salzberg, StringTie enables improved reconstruction of a transcriptome from RNA-seq reads. *Nat. Biotechnol.* **33**, 290–295 (2015).

56. S. Tang, A. Lomsadze, M. Borodovsky, Identification of protein coding regions in RNA transcripts. *Nucleic Acids Res.* **43**, e78 (2015).

57. M. A. Campbell, B. J. Haas, J. P. Hamilton, S. M. Mount, C. R. Buell, Comprehensive analysis of alternative splicing in rice and comparative analyses with Arabidopsis. *BMC Genomics* **7**, 327 (2006).

58. B. J. Haas, S. L. Salzberg, W. Zhu, M. Pertea, J. E. Allen, J. Orvis, O. White, C. R. Buell, J. R. Wortman, Automated eukaryotic gene structure annotation using EVidenceModeler and the Program to Assemble Spliced Alignments. *Genome Biol.* **9**, R7 (2008).

59. A. Marchler-Bauer, S. Lu, J. B. Anderson, F. Chitsaz, M. K. Derbyshire, C. De Weese-Scott, J. H. Fong, L. Y. Geer, R. C. Geer, N. R. Gonzales, M. Gwadz, D. I. Hurwitz, J. D. Jackson, Z. Ke, C. J. Lanczycki, F. Lu, G. H. Marchler, M. Mullokandov, M. V. Omelchenko, C. L. Robertson, J. S. Song, N. Thanki, R. A. Yamashita, D. Zhang, N. Zhang, C. Zheng, S. H. Bryant, CDD: A Conserved Domain Database for the functional annotation of proteins. *Nucleic Acids Res.* **39**, D225–D229 (2011).

60. E. V. Koonin, N. D. Fedorova, J. D. Jackson, A. R. Jacobs, D. M. Krylov, K. S. Makarova, R. Mazumder, S. L. Mekhedov, A. N. Nikolskaya, B. S. Rao, I. B. Rogozin, S. Smirnov, A. V. Sorokin, A. V. Sverdlov, S. Vasudevan, Y. I. Wolf, J. J. Yin, D. A. Natale, A comprehensive evolutionary classification of proteins encoded in complete eukaryotic genomes. *Genome Biol.* **5**, R7 (2004).

61. E. C. Dimmer, R. P. Huntley, Y. Alam-Faruque, T. Sawford, C. O'Donovan, M. J. Martin, B. Bely, P. Browne, W. M. Chan, R. Eberhardt, M. Gardner, K. Laiho, D. Legge, M. Magrane, K. Pichler, D. Poggioli, H. Sehra, A. Auchincloss, K. Axelsen, M.-C. Blatter, E. Boutet, S. Braconi-Quintaje, L. Breuza, A. Bridge, E. Coudert, A. Estreicher, L. Famiglietti, S. Ferro-Rojas, M. Feuermann, A. Gos, N. Gruaz-Gumowski, U. Hinz, C. Hulo, J. James, S. Jimenez, F. Jungo, G.

Keller, P. Lemercier, D. Lieberherr, P. Masson, M. Moinat, I. Pedruzzi, S. Poux, C. Rivoire, B. Roechert, M. Schneider, A. Stutz, S. Sundaram, M. Tognolli, L. Bougueleret, G. Argoud-Puy, I. Cusin, P. Duek-Roggli, I. Xenarios, R. Apweiler, The UniProt-GO annotation database in 2011. *Nucleic Acids Res.* **40**, D565–D570 (2012).

62. M. Kanehisa, S. Goto, KEGG: Kyoto encyclopedia of genes and genomes. *Nucleic Acids Res.* **28**, 27–30 (2000).

63. B. Boeckmann, A. Bairoch, R. Apweiler, M.-C. Blatter, A. Estreicher, E. Gasteiger, M. J. Martin, K. Michoud, C. O'Donovan, I. Phan, S. Pilbout, M. Schneider, The SWISS-PROT protein knowledgebase and its supplement TrEMBL in 2003. *Nucleic Acids Res.* **31**, 365–370 (2003).

64. S. Griffiths-Jones, S. Moxon, M. Marshall, A. Khanna, S. R. Eddy, A. Bateman, Rfam: Annotating non-coding RNAs in complete genomes. *Nucleic Acids Res.* **33**, D121–D124 (2005).

65. E. P. Nawrocki, S. R. Eddy, Infernal 1.1: 100-fold faster RNA homology searches. *Bioinformatics* **29**, 2933–2935 (2013).

66. P. P. Chan, B. Y. Lin, A. J. Mak, T. M. Lowe, tRNAscan-SE 2.0: Improved detection and functional classification of transfer RNA genes. *Nucleic Acids Res.* **49**, 9077–9096 (2021).

67. E. Birney, M. Clamp, R. Durbin, GeneWise and genomewise. *Genome Res.* **14**, 988–995 (2004).

68. S. Chen, Y. Zhou, Y. Chen, J. Gu, fastp: An ultra-fast all-in-one FASTQ preprocessor. *Bioinformatics* **34**, i884–i890 (2018).

69. B. Langmead, S. L. Salzberg, Fast gapped-read alignment with Bowtie 2. *Nat. Methods* **9**, 357–359 (2012).

70. H. Li, B. Handsaker, A. Wysoker, T. Fennell, J. Ruan, N. Homer, G. Marth, G. Abecasis, R. Durbin, 1000 Genome Project Data Processing Subgroup, The sequence alignment/map format and SAMtools. *Bioinformatics* **25**, 2078–2079 (2009).

71. A. McKenna, M. Hanna, E. Banks, A. Sivachenko, K. Cibulskis, A. Kernytsky, K. Garimella, D. Altshuler, S. Gabriel, M. Daly, M. A. DePristo, The genome analysis toolkit: A MapReduce framework for analyzing next-generation DNA sequencing data. *Genome Res.* **20**, 1297–1303 (2010).

72. B. L. Browning, Y. Zhou, S. R. Browning, A one-penny imputed genome from next-generation reference panels. *Am. J. Hum. Genet.* **103**, 338–348 (2018).

73. R. Vaser, S. Adusumalli, S. N. Leng, M. Sikic, P. C. Ng, SIFT missense predictions for genomes. *Nat. Protoc.* **11**, 1–9 (2016).

74. T. Rausch, T. Zichner, A. Schlattl, A. M. Stütz, V. Benes, J. O. Korbel, DELLY: Structural variant discovery by integrated paired-end and split-read analysis. *Bioinformatics* **28**, i333–i339 (2012).

75. L.-T. Nguyen, H. A. Schmidt, A. Von Haeseler, B. Q. Minh, IQ-TREE: A fast and effective stochastic algorithm for estimating maximum-likelihood phylogenies *Mol. Biol. Evol.* **32** 268–274 (2015).

76. X. Zheng, D. Levine, J. Shen, S. M. Gogarten, C. Laurie, B. S. Weir, A high-performance computing toolset for relatedness and principal component analysis of SNP data. *Bioinformatics* **28**, 3326–3328 (2012).

77. A. Manichaikul, J. C. Mychaleckyj, S. S. Rich, K. Daly, M. Sale, W.-M. Chen, Robust relationship inference in genome-wide association studies. *Bioinformatics* **26**, 2867–2873 (2010).

78. P. Danecek, A. Auton, G. Abecasis, C. A. Albers, E. Banks, M. A. DePristo, R. E. Handsaker, G. Lunter, G. T. Marth, S. T. Sherry, G. McVean, R. Durbin, 1000 Genomes Project Analysis Group, The variant call format and VCFtools. *Bioinformatics* **27**, 2156–2158 (2011).

79. S. Purcell, B. Neale, K. Todd-Brown, L. Thomas, M. A. R. Ferreira, D. Bender, J. Maller, P. Sklar, P. I. W. De Bakker, M. J. Daly, P. C. Sham, PLINK: A tool set for whole-genome association and population-based linkage analyses. *Am. J. Hum. Genet.* **81**, 559–575 (2007).

80. F. Abascal, A. Corvelo, F. Cruz, J. L. Villanueva-Cañas, A. Vlasova, M. Marcet-Houben, B. Martínez-Cruz, J. Y. Cheng, P. Prieto, V. Quesada, J. Quilez, G. Li, F. García, M. Rubio-Camarillo, L. Frias, P. Ribeca, S. Capella-Gutiérrez, J. M. Rodríguez, F. Câmara, E. Lowy, L. Cozzuto, I. Erb, M. L. Tress, J. L. Rodriguez-Ales, J. Ruiz-Orera, F. Reverter, M. Casas-Marce, L. Soriano, J. R. Arango, S. Derdak, B. Galán, J. Blanc, M. Gut, B. Lorente-Galdos, M. Andrés-Nieto, C. López-Otín, A. Valencia, I. Gut, J. L. García, R. Guigó, W. J. Murphy, A. Ruiz-Herrera, T. Marques-Bonet, G. Roma, C. Notredame, T. Mailund, M. M. Albà, T. Gabaldón, T. Alioto, J. A. Godoy, Extreme genomic erosion after recurrent demographic bottlenecks in the highly endangered Iberian lynx. *Genome Biol.* **17**, 251 (2016).

81. X. Zhou, F. Sun, S. Xu, G. Fan, K. Zhu, X. Liu, Y. Chen, C. Shi, Y. Yang, Z. Huang, J. Chen, H. Hou, X. Guo, W. Chen, Y. Chen, X. Wang, T. Lv, D. Yang, J. Zhou, B. Huang, Z. Wang, W. Zhao, R. Tian, Z. Xiong, J. Xu, X. Liang, B. Chen, W. Liu, J. Wang, S. Pan, X. Fang, M. Li, F. Wei, X. Xu, K. Zhou, J. Wang, G. Yang, Baiji genomes reveal low genetic variability and new insights into secondary aquatic adaptations. *Nat. Commun.* **4**, 2708 (2013).

82. R. E. Green, E. L. Braun, J. Armstrong, D. Earl, N. Nguyen, G. Hickey, M. W. Vandewege, J. A. St John, S. Capella-Gutiérrez, T. A. Castoe, C. Kern, M. K. Fujita, J. C. Opazo, J. Jurka, K. K. Kojima, J. Caballero, R. M. Hubley, A. F. Smit, R. N. Platt, C. A. Lavoie, M. P. Ramakodi, J. W. Finger Jr, A. Suh, S. R. Isberg, L. Miles, A. Y. Chong, W. Jaratlerdsiri, J. Gongora, C. Moran, A. Iriarte, J. McCormack, S. C. Burgess, S. V. Edwards, E. Lyons, C. Williams, M. Breen, J. T. Howard, C. R. Gresham, D. G. Peterson, J. Schmitz, D. D. Pollock, D. Haussler, E. W. Triplett, G. Zhang, N. Irie, E. D. Jarvis, C. A. Brochu, C. J. Schmidt, F. M. McCarthy, B. C. Faircloth, F. G. Hoffmann, T. C. Glenn, T. Gabaldón, B. Paten, D. A. Ray, Three crocodilian genomes reveal ancestral patterns of evolution among archosaurs. *Science* **346**, 1254449 (2014).

83. J. A. Robinson, D. Ortega-Del Vecchyo, Z. Fan, B. Y. Kim, B. M. vonHoldt, C. D. Marsden, K. E. Lohmueller, R. K. Wayne, Genomic flatlining in the endangered island fox. *Curr. Biol.* **26**, 1183–1189 (2016).

84. P. Dobrynin, S. Liu, G. Tamazian, Z. Xiong, A. A. Yurchenko, K. Krasheninnikova, S. Kliver, A. Schmidt-Küntzel, K.-P. Koepfli, W. Johnson, L. F. K. Kuderna, R. García-Pérez, M. de

Manuel, R. Godinez, A. Komissarov, A. Makunin, V. Brukhin, W. Qiu, L. Zhou, F. Li, J. Yi, C. Driscoll, A. Antunes, T. K. Oleksyk, E. Eizirik, P. Perelman, M. Roelke, D. Wildt, M. Diekhans, T. Marques-Bonet, L. Marker, J. Bhak, J. Wang, G. Zhang, S. J. O'Brien, Genomic legacy of the African cheetah, *Acinonyx jubatus*. *Genome Biol.* **16**, 277 (2015).

85. Y. S. Cho, L. Hu, H. Hou, H. Lee, J. Xu, S. Kwon, S. Oh, H.-M. Kim, S. Jho, S. Kim, S. Luo, W. E. Johnson, K.-P. Koepfli, A. Schmidt-Küntzel, J. A. Turner, L. Marker, C. Harper, S. M. Miller, W. Jacobs, L. D. Bertola, T. H. Kim, S. Lee, Q. Zhou, H.-J. Jung, X. Xu, P. Gadhvi, P. Xu, Y. Xiong, Y. Luo, S. Pan, C. Gou, X. Chu, J. Zhang, S. Liu, J. He, Y. Chen, L. Yang, Y. Yang, J. He, S. Liu, J. Wang, C. H. Kim, H. K. Kwak, J.-S. K. Kim, S. Hwang, J. Ko, C.-B. Kim, S. Kim, D. Bayarlkhagva, W. K. Paek, S.-J. Kim, S. J. O'Brien, J. Wang, J. Bhak, The tiger genome and comparative analysis with lion and snow leopard genomes. *Nat. Commun.* **4**, 2433 (2013).

86. H.-X. Xie, X.-X. Liang, Z.-Q. Chen, W.-M. Li, C.-R. Mi, M. Li, Z.-J. Wu, X.-M. Zhou, W.-G. Du, Ancient demographics determine the effectiveness of genetic purging in endangered lizards. *Mol. Biol. Evol.* **39**, msab359 (2022).

87. S. Feng, Q. Fang, R. Barnett, C. Li, S. Han, M. Kuhlwilm, L. Zhou, H. Pan, Y. Deng, G. Chen, A. Gamauf, F. Woog, R. Prys-Jones, T. Marques-Bonet, M. T. P. Gilbert, G. Zhang, The genomic footprints of the fall and recovery of the crested ibis. *Curr. Biol.* **29**, 340–349.e7 (2019).

88. N. F. Saremi, M. A. Supple, A. Byrne, J. A. Cahill, L. L. Coutinho, L. Dalén, H. V. Figueiró, W. E. Johnson, H. J. Milne, S. J. O'Brien, B. O'Connell, D. P. Onorato, S. P. D. Riley, J. A. Sikich, D. R. Stahler, P. M. S. Villela, C. Vollmers, R. K. Wayne, E. Eizirik, R. B. Corbett-Detig, R. E. Green, C. C. Wilmers, B. Shapiro, Puma genomes from North and South America provide insights into the genomic consequences of inbreeding. *Nat. Commun.* **10**, 4769 (2019).

89. J.-Y. Hu, Z.-Q. Hao, L. Frantz, S.-F. Wu, W. Chen, Y.-F. Jiang, H. Wu, W.-M. Kuang, H. Li, Y.-P. Zhang, L. Yu, Genomic consequences of population decline in critically endangered pangolins and their demographic histories. *Nat. Sci. Rev.* **7**, 798–814 (2020).

90. W. Yin, Z.-J. Wang, Q.-Y. Li, J.-M. Lian, Y. Zhou, B.-Z. Lu, L.-J. Jin, P.-X. Qiu, P. Zhang, W.-B. Zhu, B. Wen, Y.-J. Huang, Z.-L. Lin, B.-T. Qiu, X.-W. Su, H.-M. Yang, G.-J. Zhang, G.-M. Yan, Q. Zhou, Evolutionary trajectories of snake genes and genomes revealed by comparative analyses of five-pacer viper. *Nat. Commun.* **7**, 13107 (2016).

91. E. Palkopoulou, S. Mallick, P. Skoglund, J. Enk, N. Rohland, H. Li, A. Omrak, S. Vartanyan, H. Poinar, A. Götherström, D. Reich, L. Dalen, Complete genomes reveal signatures of demographic and genetic declines in the woolly mammoth. *Curr. Biol.* **25**, 1395–1400 (2015).

92. X. Guang, T. Lan, Q.-H. Wan, Y. Huang, H. Li, M. Zhang, R. Li, Z. Zhang, Y. Lei, L. Zhang, H. Zhang, D. Li, X. Li, H. Li, Y. Xu, M. Qiao, D. Wu, K. Tang, P. Zhao, J. Lin, S. K. Sahu, Q. Liang, W. Jiang, D. Zhang, X. Xu, X. Liu, M. Lisby, H. Yang, K. Kristiansen, H. Liu, S.-G. Fang, Chromosome-scale genomes provide new insights into subspecies divergence and evolutionary characteristics of the giant panda. *Sci. Bull.* **66**, 2002–2013 (2021).

93. Y. Ren, Q. Zhang, X. Yan, D. Hou, H. Huang, C. Li, D. Rao, Y. Li, Genomic insights into the evolution of the critically endangered soft-shelled turtle *Rafetus swinhoei*. *Mol. Ecol. Resour.* **22**, 1972–1985 (2022).

94. S. Li, B. Li, C. Cheng, Z. Xiong, Q. Liu, J. Lai, H. V. Carey, Q. Zhang, H. Zheng, S. Wei, H. Zhang, L. Chang, S. Liu, S. Zhang, B. Yu, X. Zeng, Y. Hou, W. Nie, Y. Guo, T. Chen, J. Han, J. Wang, J. Wang, C. Chen, J. Liu, J. S. Peter, M. Xu, G. Zhang, M. T. P. Gilbert, H. Yang, D. J. Erich, J. Yu, J. Yan, Genomic signatures of near-extinction and rebirth of the crested ibis and other endangered bird species. *Genome Biol.* **15**, 557 (2014).

95. X. Zhan, S. Pan, J. Wang, A. Dixon, J. He, M. G. Muller, P. Ni, L. Hu, Y. Liu, H. Hou, Y. Chen, J. Xia, Q. Luo, P. Xu, Y. Chen, S. Liao, C. Cao, S. Gao, Z. Wang, Z. Yue, G. Li, Y. Yin, N. C. Fox, J. Wang, M. W. Bruford, Peregrine and saker falcon genome sequences provide insights into evolution of a predatory lifestyle. *Nat. Genet.* **45**, 563–566 (2013).

96. W. C. Warren, D. F. Clayton, H. Ellegren, A. P. Arnold, L. W. Hillier, A. Künstner, S. Searle, S. White, A. J. Vilella, S. Fairley, A. Heger, L. Kong, C. P. Ponting, E. D. Jarvis, C. V. Mello, P. Minx, P. Lovell, T. A. F. Velho, M. Ferris, C. N. Balakrishnan, S. Sinha, C. Blatti, S. E. London,

Y. Li, Y.-C. Lin, J. George, J. Sweedler, B. Southey, P. Gunaratne, M. Watson, K. Nam, N. Backström, L. Smeds, B. Nabholz, Y. Itoh, O. Whitney, A. R. Pfenning, J. Howard, M. Völker, B. M. Skinner, D. K. Griffin, L. Ye, W. M. Mc Laren, P. Flicek, V. Quesada, G. Velasco, C. Lopez-Otin, X. S. Puente, T. Olender, D. Lancet, A. F. A. Smit, R. Hubley, M. K. Konkel, J. A. Walker, M. A. Batzer, W. Gu, D. D. Pollock, L. Chen, Z. Cheng, E. E. Eichler, J. Stapley, J. Slate, R. Ekblom, T. Birkhead, T. Burke, D. Burt, C. Scharff, I. Adam, H. Richard, M. Sultan, A. Soldatov, H. Lehrach, S. V. Edwards, S.-P. Yang, X. C. Li, T. Graves, L. Fulton, J. Nelson, A. Chinwalla, S. Hou, E. R. Mardis, R. K. Wilson, The genome of a songbird. *Nature* **464**, 757–762 (2010).

97. C.-M. Hung, P.-J. L. Shaner, R. M. Zink, W.-C. Liu, T.-C. Chu, W.-S. Huang, S.-H. Li, Drastic population fluctuations explain the rapid extinction of the passenger pigeon. *Proc. Natl. Acad. Sci. U.S.A.* **111**, 10636–10641 (2014).

98. R. B. Corbett-Detig, D. L. Hartl, T. B. Sackton, Natural selection constrains neutral diversity across a wide range of species. *PLOS Biol.* **13**, e1002112 (2015).

99. D. Le Duc, G. Renaud, A. Krishnan, M. S. Almén, L. Huynen, S. J. Prohaska, M. Ongyerth, B. D. Bitarello, H. B. Schiöth, M. Hofreiter, P. F. Stadler, K. Prüfer, D. Lambert, J. Kelso, T. Schöneberg, Kiwi genome provides insights into evolution of a nocturnal lifestyle. *Genome Biol.* **16**, 147 (2015).

100. H. Ellegren, L. Smeds, R. Burri, P. I. Olason, N. Backström, T. Kawakami, A. Künstner, H. Mäkinen, K. Nadachowska-Brzyska, A. Qvarnström, S. Uebbing, J. B. W. Wolf, The genomic landscape of species divergence in *Ficedula* flycatchers. *Nature* **491**, 756–760 (2012).

101. Y. Huang, Y. Li, D. W. Burt, H. Chen, Y. Zhang, W. Qian, H. Kim, S. Gan, Y. Zhao, J. Li, K. Yi, H. Feng, P. Zhu, B. Li, Q. Liu, S. Fairley, K. E. Magor, Z. Du, X. Hu, L. Goodman, H. Tafer, A. Vignal, T. Lee, K.-W. Kim, Z. Sheng, Y. An, S. Searle, J. Herrero, M. A. M. Groenen, R. P. M. A. Crooijmans, T. Faraut, Q. Cai, R. G. Webster, J. R. Aldridge, W. C. Warren, S. Bartschat, S. Kehr, M. Marz, P. F. Stadler, J. Smith, R. H. S. Kraus, Y. Zhao, L. Ren, J. Fei, M. Morisson, P. Kaiser, D. K. Griffin, M. Rao, F. Pitel, J. Wang, N. Li, The duck genome and transcriptome provide insight into an avian influenza virus reservoir species. *Nat. Genet.* **45**, 776–783 (2013).

102. M. L. Aslam, J. W. M. Bastiaansen, M. G. Elferink, H.-J. Megens, R. P. M. A. Crooijmans, L. A. Blomberg, R. C. Fleischer, C. P. Van Tassell, T. S. Sonstegard, S. G. Schroeder, M. A. M. Groenen, J. A. Long, Whole genome SNP discovery and analysis of genetic diversity in Turkey (*Meleagris gallopavo*). *BMC Genomics* **13**, 391–314 (2012).
